# Distinguishing features of Parkinson’s disease fallers based on wireless insole plantar pressure monitoring

**DOI:** 10.1038/s41531-024-00678-2

**Published:** 2024-03-19

**Authors:** Cara Herbers, Raymond Zhang, Arthur Erdman, Matthew D. Johnson

**Affiliations:** 1 Department of Mechanical Engineering, University of Minnesota, 111 Church Street SE, Minneapolis, 55455 MN USA; 2 Department of Biomedical Engineering, University of Minnesota, 312 Church Street SE, Minneapolis, 55455 MN USA

**Keywords:** Parkinson's disease, Parkinson's disease, Neurological manifestations, Predictive markers

## Abstract

Postural instability is one of the most disabling motor signs of Parkinson’s disease (PD) and often underlies an increased likelihood of falling and loss of independence. Current clinical assessments of PD-related postural instability are based on a retropulsion test, which introduces human error and only evaluates reactive balance. There is an unmet need for objective, multi-dimensional assessments of postural instability that directly reflect activities of daily living in which individuals may experience postural instability. In this study, we trained machine-learning models on insole plantar pressure data from 111 participants (44 with PD and 67 controls) as they performed simulated static and active postural tasks of activities that often occur during daily living. Models accurately classified PD from young controls (area under the curve (AUC) 0.99+/− 0.00), PD from age-matched controls (AUC 0.99+/− 0.01), and PD fallers from PD non-fallers (AUC 0.91+/− 0.08). Utilizing features from both static and active postural tasks significantly improved classification performances, and all tasks were useful for separating PD from controls; however, tasks with higher postural threats were preferred for separating PD fallers from PD non-fallers.

## Introduction

Postural instability is a cardinal motor symptom of Parkinson’s disease (PD) and is characterized by deficits in the control of static, reactive, and proactive posturing^1–3^. Postural instability leads to falls, loss of independence, physical injuries, and immobilization in severe cases^4^. The underlying pathophysiology of PD-related postural instability is not well understood^5^, postural instability has little or no response to current PD treatments^6,7^, and up to 90% of people with PD will fall at some stage of the disease progression^8,9^. One of the factors contributing to these challenges is a lack of quantitative, objective, and accessible approaches to effectively characterize the multi-faceted nature of postural instability in individuals with PD.

The clinical gold standard of postural instability assessments is the retropulsion test, which involves pulling back on an individual’s shoulders and visually inspecting how many steps are necessary to regain balance^10^. While well-validated, the retropulsion test is subjective, requires a trained clinician, and addresses only reactive balance, which is just one aspect of postural control^11^. Several studies have demonstrated that monitoring the center of pressure (COP) over a variety of balance tasks is a useful and objective way to identify altered postural control in PD^12,13^, especially in individuals with PD who have a history of falls^14,15^. These COP measurements are especially valuable for assessing balance tasks involving heightened postural threat, which is characterized by significant disturbances to the postural control system, prompting individuals to adjust their posture to maintain an upright position^16^. An increase in postural threats can be due to things such as: (1) removing visual cues while maintaining an upright stance, (2) changing the support surface from a hard flat surface to a soft, tilted surface, or (3) removing a traditional base of support by requiring an individual to stand upright with only one foot on the ground as opposed to both feet. However, traditional methods to assess COP during a variety of balance tasks are not widely available outside of research settings and therefore are not useful in many clinical or at-home settings.

Monitoring insole plantar pressure^17^ could be a simple, low-cost, and accessible way to frequently assess postural control through COP measurements in each foot. However, studies demonstrating that plantar pressure data can be used to identify PD-related postural instability are limited. In a small feasibility study, plantar pressure was used to identify PD-specific balance patterns during quiet stance and gait^18^. Other studies have shown that plantar pressure can predict gait dysfunction in PD^19,20^, but these studies did not assess PD-related postural instability.

In this study, we leveraged (1) previous findings showing features of COP are different in individuals with PD and in PD with a fall risk^21,22^, (2) the development of insole pressure sensors as a convenient way to collect COP outside of the clinic, and (3) careful selection of balance tasks that engage different aspects of one’s postural control system to train a series of machine-learning models for detection of PD and PD who have a history of one or more falls. We hypothesized that utilizing both static and active balance tasks would improve classification performance between control and PD subjects. Previous work has also demonstrated that tasks with higher postural threat help with identifying individuals with PD who are at risk for falling^14,23–25^. We further hypothesized that models would put more weight on active balance task features to differentiate PD fallers from PD non-fallers.

## Results

**Fig. 1:**
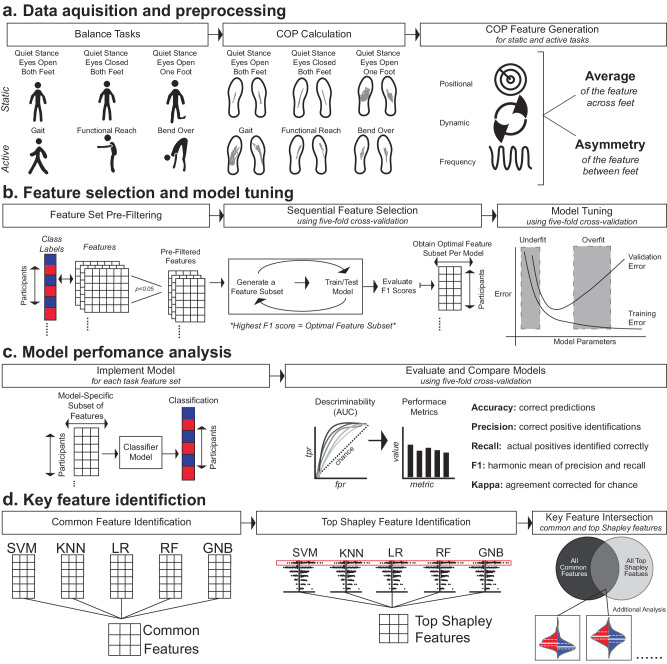
Machine-learning models were developed to classify PD from young controls, PD from age-matched controls, and PD fallers from PD non-fallers, based on features derived from insole plantar pressure. **a** Plantar pressure was recorded and used to calculate COP per foot from three static balance tasks and three active balance tasks. Positional, dynamic, and frequency domain features were generated from the COP. The average of each feature and the asymmetry of each feature across feet were computed. The static task and active task features were utilized separately and combined to develop the machine-learning classifier models. **b** *F*-statistic pre-filtering and forward sequential feature selection were used to identify an optimal subset of features which maximized each model’s five-fold cross-validation F1 score. Once the subset of features per model was determined, hyperparameter tuning over a five-fold cross-validation was completed. **c** From the tuned model, the model performance was assessed, and individuals who were misclassified across models were identified. **d** Features commonly chosen by feature selection methods were identified, and Shapley analysis was performed for each model to identify features with the highest Shapley value. Features that appeared in the common feature set and top Shapley value feature set were analyzed to understand their underlying biomechanical implications related to PD and individuals with PD who have a history of falls.

Insole plantar pressure was collected while subjects performed three static balance tasks (quiet stance eyes open, quiet stance eyes closed, quiet stance eyes open on one foot) and three active balance tasks (gait, functional reach, bending over) (Fig. 1). These data were collected across varying participant demographics and surface types (Supplementary Table 1). After collection of insole plantar pressure data, COP-derived features collected during these tasks served as input to train five machine-learning model architectures (SVM: support vector machine, RF: random forest, LR: logistic regression, KNN: K-nearest neighbors, and GNB: Gaussian naive Bayes) to differentiate between PD subjects and controls (young and age-matched), and between individuals with PD who do and do not have a history of falls (PD fallers and PD non-fallers, respectively). COP-derived features from static and active tasks were used separately and then together. For each classification, the feature sets were first pre-filtered by way of *F*-statistic feature selection. Then, forward sequential feature selection with five distinct machine-learning model architectures was used to determine an optimal subset of features that maximized each model’s classification performance, measured by the five-fold cross-validation F1 score. After determining the optimal feature subset, the hyperparameters of each model were tuned, and the performance metrics of each model were determined using a five-fold cross-validation. Complete model performance results and all performance metrics are shown in Supplementary Figs. 1–6.

### COP-derived feature differences between PD and control subjects

**Fig. 2:**
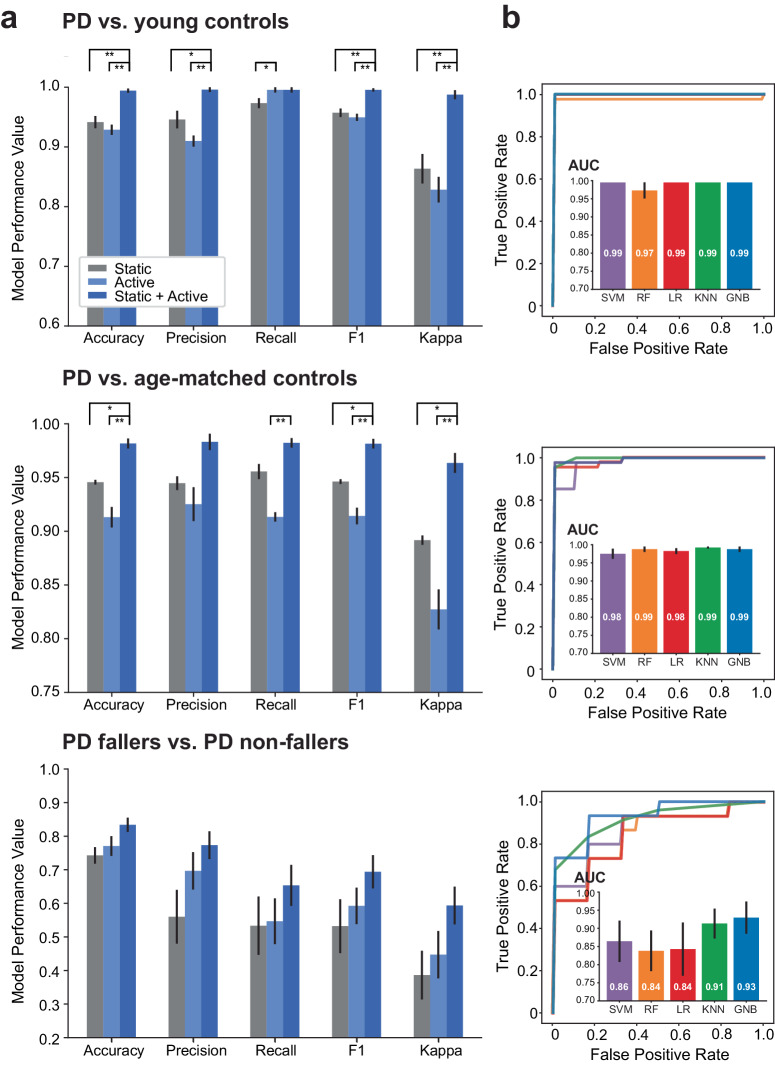
Model performance for all three binary classifications. **a** Average model performance (with standard error mean) for models trained on static task features only, active task features only, and static+active task features. Models trained on static+active task features demonstrated the highest classification accuracies. **b** The average ROC curves and associated AUC (with standard error mean) based on five-fold cross-validation are shown for all models trained on static+active task features. **p < 0.001, *p < 0.017. (SVM support vector machine, RF random forest, LR logistic regression, KNN *K*-nearest neighbors, GNB Gaussian naive Bayes).

For comparisons of COP between PD subjects and young controls (Fig. 2a) and between PD subjects and age-matched controls (Fig. 2b), there was no effect significant effect of model type on classification. Additionally, models trained on only static or only active tasks performed similarly across all metrics. In contrast, the models trained on static+active task features performed significantly better than the models that were trained on only static task features (young controls: accuracy *p* < 0.001, precision *p* = 0.0011, f1 *p* < 0.001, kappa *p* < 0.001; age-matched controls: accuracy *p* = 0.008, f1 *p* = 0.007, kappa *p* = 0.008) and models trained only on active task features (young controls: accuracy *p* < 0.001, precision *p* < 0.001, f1 *p* < 0.001, kappa *p* < 0.001; age-matched controls: accuracy *p* < 0.001, recall *p* < 0.001, f1 *p* < 0.001, kappa *p* < 0.001). Models trained on features from the static+active tasks that best classified PD from young control groups, based on five-fold cross-validation performances, were the support vector machine, *K*-nearest neighbors, and Gaussian Naïve Bayes models (AUC of 0.99+/− 0.00 [standard error mean]; Fig. 2a and Supplementary Figs. 1 and 4). For classification between PD and age-matched control groups trained on static+active task features, again based on five-fold cross-validation performances, the *K*-nearest neighbors model performed the best (AUC of 0.99+/− 0.01 standard error mean; Fig. 2b and Supplementary Figs. 2 and 5).

### COP-derived feature differences between PD fallers and non-fallers

When comparing PD fallers with non-fallers, there was no significant effect on model type for any performance metrics. There was a marginal improvement in classification performance with models trained on active task features compared to static task features but with no significant difference. Similarly, there was a marginal improvement in classification performance for models trained on static+active task features compared to only static task features or only active task features, but no significant differences (Fig. 2c). The model that best classified between PD faller and PD non-faller groups, based on five-fold cross-validation performances, was the Gaussian Naïve Bayes model. This model achieved an AUC of 0.93 + /− 0.04 (standard error mean) (Fig. 2c and Supplementary Figs. 3 and 6).

### Common features

**Fig. 3:**
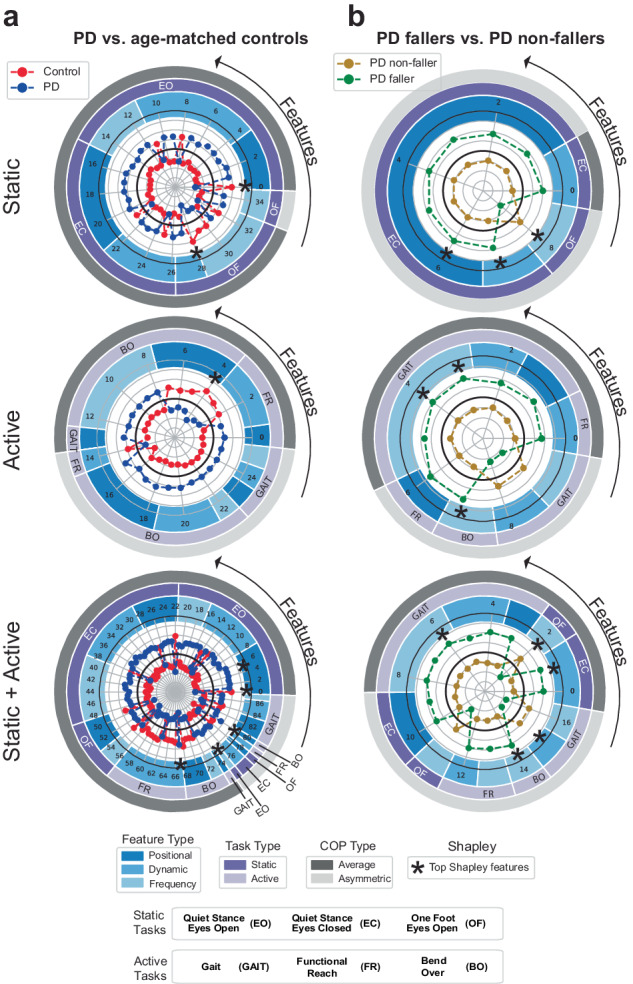
Common features chosen by at least three of the five models during feature selection. **a** PD and age-matched controls, and **b** PD fallers and PD non-fallers. Common features were identified for models trained on static task features only, active task features only and static+active task features. The *z*-score of each feature, per group, is shown in the polar plot. The inner black circle indicates a *z*-score of zero, with radius increments of 0.25. The surrounding rings indicate each feature’s domain, task type, and COP type. Features that also had the single highest Shapley value in a model are also indicated.

When comparing data from individuals with PD and age-matched controls, models trained on the static task shared 35 common features, derived mostly from the quiet stance with eyes open task and averaging foot COP data (Fig. 3a and Supplementary Fig. 8a). For models trained on the active task, 26 features were common with most from the bending-over task and an even split between average and asymmetric COP features. Combining static and active task features resulted in 88 common features, mostly from static balance tasks, and predominantly average features. Among the common features, 7 unique features also had top Shapley values.

For data collected between PD fallers and PD non-fallers, models trained on the static task had nine common features, derived primarily from the quiet stance eyes closed task and from asymmetric COP foot metrics (Fig. 3b and Supplementary Fig. 8b). For models trained on active task features, there were 10 common features, derived mostly from the gait task. For models trained on static+active task features, there were 17 common features, again derived primarily from the gait task. Out of the common features for static, active, and static+active models, 9 unique top Shapley features were identified.

### Key features

**Fig. 4:**
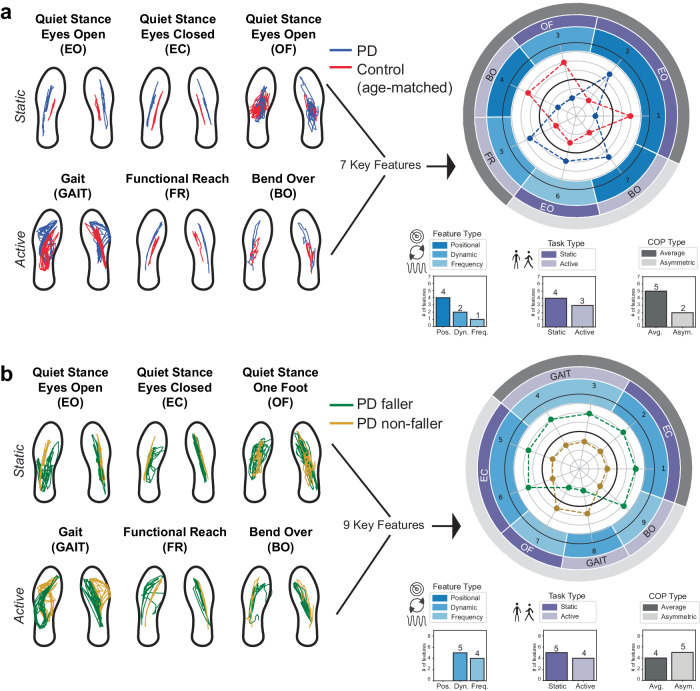
Key features appearing as both common features and top Shapley features. COP throughout the duration of each task was used to generate a set of features, which were leveraged to train and test five machine-learning model architectures to classify between **a** individuals with PD and age-matched controls, and between **b** PD fallers and PD non-fallers. Common features amongst the models during feature selection with the single highest Shapley value in at least one model are shown with *z*-score values for each feature, per group, in the polar plot. See the Fig. 3 caption for more details. Feature numbers correspond to rows in Table 1.

For models comparing individuals with PD and age-matched controls, 7 features that were common across static, active, and static+active tasks also appeared as top Shapley features in a model (Fig. 4a and Table 1). Four were from static balance task features (3 eyes open, 1 one foot) and three were features from active balance tasks (2 bend over, 1 functional reach). No features appeared from gait tasks. Five were positional measures, one was a dynamic measure, and one was a frequency measure (Supplementary Fig. 11a).

**Table 1:** List of key features at the intersection of top Shapley and common features for classifications between (top) individuals with PD and age-matched controls, and (bottom) PD fallers and PD non-fallers

Feature #	Feature (COP type, task, feature name)
**Key features separating: PD from age-matched control subjects**	
1	Average, eyes open, mean value ML
2	Average, eyes open, rms radius
3	Average, one foot, mean frequency ML
4	Average, bend over, mean value ML
5	Average, functional reach, mean peak sway density
6	Asymmetric, eyes open, energy content between 0.5–2 Hz PSD ML
7	Asymmetric, bend over, mean value ML
**Key features separating: PD fallers from PD non-fallers**	
1	Average, eyes closed, zero crossing velocity ML
2	Average, eyes closed, zero crossing velocity AP
3	Average, gait, energy content below 0.5 Hz PSD ML
4	Average, gait, total power PSD ML
5	Asymmetric, eyes closed, confidence ellipse area
6	Asymmetric, eyes close, mean distance peak sway density
7	Asymmetric, one foot, frequency mode PSD AP
8	Asymmetric, gait, sway area per second ML and AP
9	Asymmetric, bend over, power frequency 50% PSD AP

Additionally, for models trained to differentiate PD fallers from non-fallers, 9 features that were common across static, active, and static + active tasks models also appeared as top Shapley features in a model (Fig. 4b and Table 1). Five were static balance task features (4 eyes closed, 1 one foot), and four were from active balance tasks (3 gaits, 1 bend over). No features appeared from a quiet stance eyes open or from functional reach. Further, five were dynamic features and four were frequency features. There were no positional features. Lastly, four of the features were average features and five were asymmetric features (Supplementary Fig. 11b).

## Discussion

This study demonstrates the utility of insole plantar pressure recordings during simulated daily living activities to differentiate between individuals with PD and control subjects (young and age-matched), as well as between PD fallers and PD non-fallers. Feature selection and Shapley analysis of the machine-learning models further enabled the identification of specific tasks (static and active), COP trajectories (position, dynamic, and frequency), and consistency of COP between feet (average and asymmetry) that best differentiated between groups.

This study investigated binary classification models leveraging insole pressure data from static and active tasks to differentiate PD subjects from young and age-matched control groups. The classifications assessed in this study achieved high performance (with F1 scores greater than 97% for all models trained on static+active features). Additionally, three models classifying PD from young controls (support vector machine, K-nearest neighbors, and Gaussian Naïve Bayes) demonstrated perfect classification (100% accurate) over a five-fold cross-validation. Together, these data demonstrate clear decision boundaries from insole plantar pressure-derived features that separate PD and control subjects when utilizing multiple simulated tasks that are often performed as part of daily living.

Previous studies that have leveraged machine-learning models to classify PD and control groups have focused primarily on one task, including quiet stance (86% accuracy)^26^ or gait (95% accuracy)^27^. The improved performance achieved by static+active tasks models in this study likely stems from choosing tasks that sample non-redundant aspects of postural control. This finding emphasizes the differences in the manifestation of postural control dysfunction amongst individuals with PD and demonstrates the clinical need to quantify a variety of balance assessments to fully capture the high dimensionality of PD-related postural instability.

In conjunction with combining static+active tasks in the models, the use of insole plantar pressure from each foot enabled assessment of COP asymmetry, which proved helpful in differentiating PD fallers from PD non-fallers. In this study, the KNN model trained on insole plantar pressure features from both static and active tasks, achieved 91% accuracy, 93% precision, and 80% recall. A previous study using a force plate to capture COP metrics during static balance tasks and a decision tree model had performance results of 88% accuracy, 75% precision, and 57% recall^24^. Other studies have investigated COP characteristics to differentiate between controls, PD non-fallers, and PD fallers, but did not use insole plantar pressure sensors, machine-learning algorithms, or investigate the effect of balance task type on classification^23,25^.

This study also investigated the effect of task type on model classification performance between PD fallers and PD non-fallers. Models using active task features showed slightly better performance than those using static task features, and models trained on both static+active features showed the highest performance. Abnormalities in postural control for PD fallers have been shown to be more evident during active tasks^14^ and static tasks with induced postural threat^28^, explaining why models using active tasks performed better, and static+active task models had the best classification performance. These findings highlight the importance of using a variety of balance assessments to fully capture the high dimensionality of postural instability, especially tasks with higher postural threat to assess fall risk in PD.

Using common feature identification and Shapley analysis, the models identified seven key features that had a high marginal contribution to classifying subjects with PD from age-matched controls. While these features were biomechanically relevant, classification performance was higher when additional features (beyond the seven) were methodically included in the model’s input. Therefore, these seven features were not sufficient on their own to classify between groups, but their biomechanical relevance was strongly relevant to the overall classification performance.

The ankle and hip joints are the primary contributors to anteroposterior (AP) and mediolateral (ML) COP displacement, respectively, during postural control^29,30^. In individuals with PD, ankle joint torques are decreased, leading to compensatory mechanisms such as increased hip joint torques^31^. This compensation was observed in the key feature set of the study, as individuals with PD exhibited an increase in the lateral placement of weight on each foot during quiet stance with eyes open and bending over. Since individuals were instructed to place their feet at a comfortable width, as opposed to a normalized stance width, this key feature may provide insight into individuals with PD electing to take a stance width that emphasizes lateral weight distribution, or simply an attempt to put more weight on the outside of their feet to increase the area of base support or compensate for decreased ankle joint control.

On the contrary, the mean frequency ML was decreased in individuals with PD compared to age-matched controls during a quiet stance on one foot. This may be counterintuitive, as an increase in this feature may be indicative of more ML sway in order to maintain balance^32^. However, during quiet stance one foot, individuals with PD more often placed their non-support foot on the ground to regain balance, while controls were able to maintain upright on one foot for longer (see Supplementary Fig. 12). Therefore, the increase in ML sway in controls may be representative of an increased postural control threshold to maintain balance on one foot, through ML sway compensation, as opposed to individuals with PD who had to compensate for a decreased threshold and instead placed their non-support foot temporarily on the ground to maintain upright posture.

Additionally, the rms of the radius of COP during quiet stance with eyes open was higher in individuals with PD. This feature reflects the distance covered by the AP and ML components of COP, and was shown to be on average larger in PD as compared to controls. There was also a large distribution of this feature seen in individuals with PD as compared to controls. These findings align with previous studies that found machine-learning algorithms could classify PD subjects from control subjects based on postural sway features^26,33^. Specifically, postural sway was greater and more variable in PD subjects compared to controls^26^, and that rms of the COP was a useful feature in the differentiation^33^.

The mean peak sway density during functional reach was higher between individuals with PD compared to controls. This feature measures the consecutive samples of the COP trajectory that, for each instant, fall within a 3 mm circle. For static tasks, high peak sway density corresponds to stable postural control^34^. However, for active tasks such as functional reach, as shown in this study, the PD group demonstrated a larger mean peak sway density than controls. While perhaps counterintuitive, the increase may demonstrate a more rigid or slower movement over the duration of the active task for the PD group in comparison to controls. Further assessment of this feature should be evaluated to determine whether its biomechanical underpinnings are due to increased rigidity or bradykinesia^35,36^.

Features that represented an increase in asymmetric postural control in PD were also highlighted by the models. The asymmetric energy content between 0.5 and 2 Hz ML during quiet stance with eyes open and the asymmetric mean value ML during bending over was increased in individuals with PD compared to controls. Asymmetric posture is due to larger joint torques from one side of the body as opposed to the other^37^. For these features, it is likely that the asymmetry occurred because one hip joint produced more hip torque than the other, which resulted in asymmetric ML features across the feet. This aligns with previous findings that have demonstrated that asymmetric postural control occurs in PD^38^, which may be further influenced by the asymmetric presentation of other PD motor signs^39^.

Within the PD cohort, nine unique features strongly influenced the classification between PD fallers and PD non-fallers. The tasks underlying these nine features in the machine-learning models were either active tasks or static tasks with induced postural threat by way of removing visual cues (eyes closed) or reducing the base of support (standing on one foot). Previous studies have also observed that active tasks and static tasks with induced postural threat result in different postural control responses between PD fallers and PD non-fallers^14,23–25^. The average zero crossing in the ML and AP directions, defined as the number of times the COP velocity crosses the zero-value axis, was higher in PD fallers. Previous work has shown an association between this variable and people in general who fall^40^, but this metric may also prove useful for predicting PD fallers from PD non-fallers.

The average energy content below 0.5 Hz power spectral density (PSD) ML and total power PSD ML during gait was larger in PD fallers compared to PD non-fallers. This may indicate that there is more postural sway in the ML direction during gait for PD fallers, or that PD fallers deviate from a straight walking path more often, or a combination of both. This aligns with previous findings, which saw increased ML head and hip motion in those at higher risk of falls with PD^41^. Additionally, greater postural sway in the ML direction stance has been associated with an increased risk of falls in individuals with PD^42,43^. Increased postural sway during gait may be due to impaired postural responses in the ankle and hip, or evidence of cognitive compensation due to decreased gait automation^44,45^.

During quiet stance with eyes closed, the models showed that PD fallers have increased asymmetry in their confidence ellipse area as well as their mean peak sway density. Similarly, increased asymmetry was observed in power frequency 50% PSD AP during the bending over task. Asymmetric posture can stem from larger joint torques from one side of the body^37^; however, in this study, it was also seen that PD fallers exhibited decreased asymmetry in two other key features: quiet stance one-foot frequency mode PSD AP and gait sway area per second ML and AP. While previous findings have demonstrated that asymmetric postural control occurs in PD^38^, there are mixed results as to whether PD fallers exhibit more or less postural asymmetry in comparison to PD non-fallers^46,47^. Beretta et al. showed that COP asymmetry increased in PD fallers and could be used in a machine-learning model to classify PD non-fallers from PD fallers^46^. In contrast, Barbieri et al. demonstrated that PD fallers had decreased asymmetry in their postural control^47^.

PD symptoms typically present unilaterally (and therefore asymmetrically), and this may contribute to the asymmetric posture observed in PD^48^. However, as the disease progresses, the symptoms often become bilateral, which may decrease the asymmetric nature of PD-related postural instability. The transition from unilateral to bilateral symptom presentation may be why there have been mixed results as to whether or not PD fallers exhibit decreased or increased asymmetric postural control. Similarly, the heterogeneous presentation of PD and the non-linear mixing of symptoms may also contribute to these results. One advantage of the insole plantar pressure measurement approach is that such questions could be followed up upon through longitudinal tracking of subjects in their activities of daily living over time.

This study demonstrated the differential utility of insole plantar pressure sensor data in the context of people with and without PD, and with and without a history of falls in the context of PD, suggesting a potential approach for telehealth tracking of postural instability. The wearable sensors are relatively low-cost and accessible within multiple contexts (Supplementary Table 1), and the brief balance tasks can be completed in under 10 min and do not require a trained physician. The low barrier of entry presents opportunities for insole sensors to frequently and objectively assess fall risk in PD, with falls and the fear of falling often contributing to significant reductions in quality of life.

Additionally, this study showed that interpretable, quantitative COP measurements can be used in machine-learning models to identify individuals with PD and those with a history of falls. Although interpretable COP features were used, it was not necessary to understand every input feature to comprehend the model’s output. This balance between model simplicity and interpretability can directly benefit clinicians and patients by identifying postural instability severity and indirectly impact future treatments for PD-related postural instability by providing further insight into this motor symptom.

In the context of interpreting the results from this study, it is important to consider several limitations. First, portions of the dataset were imbalanced, with a higher percentage of participants in the PD group than in the young control group, and in the PD non-faller group than the PD faller group. Second, data were collected in varying locations, with significant differences in surface type between PD and age-matched control groups. While this limitation introduced additional variability in the study, it also demonstrated that data collection location did not impede the classifier models and showed the robustness of the insole plantar pressure approach across varying support surfaces. Third, there was a significant difference in sex between the age-control and PD groups. Therefore, while the features provide insight into differences between the posture of PD and age-matched control groups, sex is a confounding factor that may also influence these features.

This study establishes and reinforces important insole plantar pressure features across tasks that could be used for improved tracking of PD-related postural instability progression and more effective evaluation of patient-specific treatments in the future. To address the identified limitations, the next steps should be considered to further refine how plantar pressure features provide insight into individuals with PD who fall. To begin, future studies should consider expanding the size of the dataset to (1) further validate current classification models and key features with a new cohort, (2) capture more edge cases of PD for improved model classification, (3) enable additional comparisons, like distinguishing between age-matched fallers and PD fallers, and (4) minimize confounding differences between some of the group comparisons, such as sex or surface type. Similarly, comparing classification model outcomes to clinically relevant ratings of PD fall risk, such as the Unified Parkinson’s Disease Rating Scale or Berg Balance Scale, would provide valuable insights and guidance for the use of the identified features within telehealth contexts and within contexts to quantify the degree of postural instability.

While this study demonstrated the usefulness of capturing COP in the context of various balance tasks, future work should further refine how plantar pressure features provide insight into individuals with PD who fall. Falls in PD have been shown to be closely related to dysfunction in gait^41^, and many of these gait dysfunctions can be captured from metrics such as stride length, stride width, swing time, and stance time^49^. Future studies should consider leveraging the insole plantar pressure sensors, likely coupled with wearable inertial measurement units, to extrapolate these important gait metrics and test whether their inclusion would provide greater classifier performance for individuals with PD who are at risk for falling. Similarly, instability characteristics of PD have been shown to be exacerbated during turning and dual-task gait (i.e., when an individual performs a gait task while also completing a cognitive task, such as counting backward from one hundred by threes)^50^. Subsequent studies with the insole plantar pressure sensors may consider including such tasks, as they hold the potential to offer deeper insights into the vulnerability of falls among individuals with PD. Although participants performed turns within the scope of this study’s gait task, the event-based analyses did not enable isolating specific nuances of turning movements.

Lastly, the at-home use of insole plantar pressure systems for assessing fall risk in PD will require technical developments^51^. One could envision processing the data collected during a brief set of tasks, uploading it to the cloud through a smartphone or tablet, and then using the pre-trained machine-learning models to map the plantar pressure data to forecast falling risk across tasks, environments, and time. Most studies tracking the progression of postural instability in PD have only sampled on the order of several months to years^21,52,53^, and little is known if ultradian, circadian, and infradian rhythms of postural instability are present as people with PD go about their daily activities^54^. This is an important next step to track fall risk longitudinally and on an individual basis.

Machine-learning models, based on insole plantar pressure data collected during six simple static and active postural control tasks, successfully differentiated between people with and without PD, and between PD fallers and PD non-fallers. The COP-derived feature input to the model was biomechanically relevant and interpretable, showing how specific static and active tasks were useful for differentiating PD and how tasks with greater postural threat were useful for differentiating PD fallers from PD non-fallers. The approach points toward an objective, at-home tool for rapid and longitudinal assessment of PD-related postural instability.

## Methods

### Participants

One hundred eleven people were recruited for this study: 44 were diagnosed with PD and 67 were control subjects (Table 2). The 44 oldest subjects were considered age-matched controls, and the remaining 23 were considered young controls. There was no significant difference between ages for PD and age-matched control subjects (Welch’s *t*-test, *p* = 0.09). The inclusion criteria for participants were that they were able to complete the balance tasks and that they were over the age of 18. Controls could not have any form of neurological condition that affected movement, and individuals with PD needed to self-report a diagnosis of PD as assessed by their treating physician and not have any other form of neurological condition that affected their movement. The purpose of this criteria was to exclude other motor control disorders from the study. Data were collected while the PD subjects were on their typical medication and/or deep brain stimulation regimen. After providing informed consent, all participants completed a questionnaire with information on sex, race, age, and fall history. At the time of self-reporting fall history, individuals were asked whether they had experienced a fall within the past year. Individuals who had experienced at least one fall in the past year were considered fallers. PD participants completed an additional questionnaire that provided information on PD duration when medication was last taken, and whether they had an implanted deep brain stimulation system (Table 2). Information regarding each participant’s PD diagnosis, medication and deep brain stimulation state, fall history, and associated demographics were self-reported and not accompanied by clinical assessments by a trained neurologist. The study was approved by the University of Minnesota Institutional Review Board (IRB Study Number: STUDY00013580). All subjects gave written, informed consent prior to participation.

**Table 2:** The demographics of participating subjects in this study given by their mean (standard deviation)

	Young controls	Age-matched controls	PD
Sample size	23	44	44
Age, in years	28.9 (5.2)	59.0 (10.2)	65.7 (7.9)
Years with PD	NA	NA	8.2 (5.2)
Deep brain stimulation implant (% yes)	NA	NA	20%
Sex (% male)	47.8%	45.5%	68%^a^
Self-reported balance issues (% yes)	0.09%	15.9%	77.3%
Self-reported faller (% yes)	0%	0%	34.1%

See Supplementary Table 1 for information on data collection sites and surfaces.

^a^A significant difference in sex was observed between PD and age-matched control groups (*p* = 0.03), which is consistent with a higher prevalence of PD in males.

After completing the questionnaire, all participants were instructed to complete a series of static and active balance tasks while wearing insole plantar pressure sensors (see below). Each task was completed one time, per participant. The tasks were completed outside of a traditional lab-based environment, and instead in the context of a common location that the participant goes to in their daily life. Therefore, the location of data collection varied across participants. Due to varying locations where testing took place, the surface that the subject stood on also varied. The types of surfaces used in this study included hardwood floors, pavement, concrete, carpet, and turf (Supplementary Table 1).

Balance tasks were categorized into three static tasks and three active tasks. Tasks that required the individual to maintain a still posture were categorized into static tasks^1^. Tasks that required the individual to initiate a voluntary movement to complete the task were categorized into active tasks^1^. These tasks were chosen based on previous research that has assessed postural control in PD, tasks included in the MDS-UPDRS assessments of movement, tasks included in the Berg Balance Scale, and postural tasks that are commonly completed during daily life^55–59^. Both static and active tasks were included because they recruit different aspects of the postural control system^60^.

### Static balance tasks

Static postural control is defined as achieving postural alignment while minimizing movement of body segments, which requires a complex state of sensorimotor control loops that contribute to balance control^61^. The goal of static postural control is to maintain the upward alignment of the body so that the effect of gravitational forces that displace the body’s center of mass can be minimized. The first static task required the participant to stand still for 30 s with their eyes open (EO: quiet stance eyes open). The next static task required the participant to stand still for 30 s with their eyes closed (EC: quiet stance eyes closed). For both eyes open and eyes closed static tasks, stance width was not normalized; instead, participants were instructed to place their feet at a comfortable distance apart. The last static task required the participant to stand on their left foot for 30 s then stand on their right foot for 30 s each with their eyes open (OF: quiet stance one foot). Each participant was allowed to put their non-standing foot down to regain balance if needed but was required to return to a one-legged stance immediately afterward.

### Active balance tasks

Active postural control requires the ability to remain stable while performing voluntary movements or actions that include movement from an individual’s initial body placement^62^. These active movements require the integration of many sub-components which ultimately result in coordinated and steady movements. These sub-components include proprioception, the range of motion of lower limb joints, the strength of muscles, automatic reflexes, and movement cognition. The first active task required the participant to walk 3 meters, turn, and walk back to their starting location (GAIT: gait). The next active task required the participant to stand normally and then reach both arms forward as far as possible without losing balance. Once the reach limit was hit, the participant was instructed to return back to their normal upright stance (FR: functional reach). For the last active task, the participant was instructed to bend down and pick up an item off the ground that was placed two feet in front of them. The participant was allowed to bend their knees while picking up the object (BO: bend over).

### Data processing

**Fig. 5:**
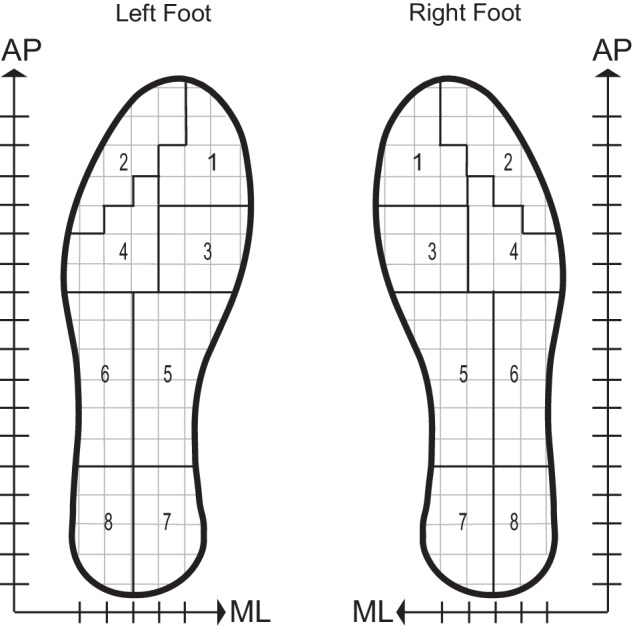
The plantar pressure sensors collected force data at each sensor in the insole. The average force value from each of the eight anatomical areas was used for COP calculation. Coordinates were defined by the distance between arrays in the matrix, marked by dashed lines on the mediolateral (ML) and anteroposterior (AP) axes.

During all tasks, plantar pressure data was collected using insole plantar pressure sensors (PRISM, RetiSense, India). The participants wore properly fitting athletic shoes with the proper sized insole sensor inside each shoe. Each pressure insole contained 100 sensors distributed as a matrix array. The RetiSense PRISM sensors collected force data at each sensor in the insole. During data collection, the back end of the RetiSense software averaged the data from the 100 pressure sensors into eight groups, which were determined by anatomically relevant locations: big toe (area 1), smaller toes (area 2), metatarsal 1 and 2 (area 3), metatarsal 3 and 4 (area 4), medial arch (area 5), lateral arch (area 6), medial heel (area 7), and lateral heel (area 8). Coordinates were defined by the distance between arrays in the matrix, marked by dashed lines on ML and AP axes (Fig. 5).

The raw data from the plantar sensors comprised of the force values across the eight locations of each foot and a time stamp for each sample. For 30 controls and 43 PD subjects, the data were collected with a sampling rate of 50 Hz. For 37 controls and 1 PD, the data were collected with a sampling rate of 12 Hz. Based on comparisons between sampling rates, the data collected at 50 Hz was filtered with an IIR anti-aliasing filter and down-sampled to 12 Hz for consistency.

### Center of pressure calculation

The force data were post-processed to calculate the COP for each foot at each sample. The COP at a moment in time was defined as an ML and AP coordinate of the average location based on all pressure acting upon the insole. COP was calculated for each foot separately by multiplying each force value, ${F}_{i}$, by the ML distance ${(D}_{{ML}})$ and AP distance ${(D}_{{AP}})$ from the insole origin and dividing by the total force summation (Eqs. 1–2, Supplementary Table 2).

1 $${{COP}}_{{ML}}=\frac{{\sum }_{n=1}^{8}{F}_{i}\,\cdot\, {D}_{{ML}}}{{\sum }_{n=1}^{8}{F}_{i}}$$

2 $${{COP}}_{{AP}}=\frac{{\sum }_{n=1}^{8}{F}_{i}\,\cdot\, {D}_{{AP}}}{{\sum }_{n=1}^{8}{F}_{i}}$$

### Feature set generation

The time-varying COP signal for each foot was measured throughout the duration of each task, and then used to calculate 60 features^63^ per foot for each task completed (Supplementary Tables 3–5). The 60 features were further categorized into positional, dynamic, and frequency features. Positional features describe characteristics of the dispersion of the trajectory or position of the feet and do not require the knowledge of the dynamics of the signal. Nineteen positional features were used (Supplementary Table 3). Dynamic features are based on the dynamic of the COP, requiring the knowledge of its local displacements. Twenty-one dynamic features were used (Supplementary Table 4). Frequency features are used to describe the power spectral density of the COP trajectory. Twenty frequency features were used (Supplementary Table 5). These features were calculated using open-source code^63^, which provides details on the original formulations. Positional, dynamic, and frequency features were calculated for each task and foot, and the features were then averaged across both feet to determine an average for each feature (Eq. 3). Asymmetry of the features between feet was also calculated as the absolute difference of features, relative to the sum of the features from each foot (Equation 4). This resulted in 60 average features and 60 asymmetric features per participant per task, for a total of 120 features per participant and per task. For the one-foot stance task, only the features from the weight-bearing foot were considered. Normalization of the features was done using a minimum-maximum scaler between zero and one.

3 $${{{\mathrm{feature}}}}_{{{\mathrm{avg}}}}=\frac{{{{\mathrm{feature}}}}_{{{\mathrm{leftfoot}}}}+{{{\mathrm{feature}}}}_{{{\mathrm{rightfoot}}}}}{2}$$

4 $${{{\mathrm{feature}}}}_{{{\mathrm{asym}}}}=\frac{\left|{{{\mathrm{feature}}}}_{{{\mathrm{leftfoot}}}}-{{{\mathrm{feature}}}}_{{{\mathrm{rightfoot}}}}\right|}{{{{\mathrm{feature}}}}_{{{\mathrm{leftfoot}}}}+{{{\mathrm{feature}}}}_{{{\mathrm{rightfoot}}}}}$$

Features were used to train binary machine-learning classifiers to classify (1) young control from PD participants, (2) age-matched control from PD participants, and (3) PD non-fallers from PD fallers. The young controls vs. PD classifier assessed the feasibility of identifying PD based on PD-specific balance patterns. The age-matched control vs. PD classifier investigated whether PD could be identified based on altered postural control while reducing age-related confounding factors. The PD non-fallers vs. PD fallers classification aimed to identify different severities of PD-related postural instability (non-faller being less severe postural instability, and faller being more severe postural instability). The static and active task feature sets were used separately and together (static, active, and static+active) to investigate the influence of task type on classification performance. To investigate potential confounding factors, exploratory analysis (Welch’s *t-*test) was conducted to compare surface types, sex, and age between groups (age-matched controls vs. PD and PD non-fallers vs. PD fallers). A significant difference was defined at *p* < 0.05.

### Feature selection and model development

Before developing the machine-learning classifier models, the static, active, and static+active feature sets were each pre-filtered and sub-selected to determine an optimal subset of features (see below). These optimal sets of features were then used to train five different binary classification machine-learning architectures: support vector machine (SVM), random forest (RF), logistic regression (LR), K-nearest neighbors (KNN), and Gaussian naive Bayes (GNB). Python’s sci-kit learn package was used for the five machine-learning model architectures (SVM: sklearn.svm.SVC, RF: sklearn.ensemble.RandomForestClassifier, LR: sklearn.linear model.LogisticRegression, KNN: sklearn.neighbors.KNeighborsClassifier, GNB: sklearn.naivebayes.GaussianNB)^64^.

### Feature set pre-filtering

In order to prune the feature space used to train the models, an F-statistic filtering method was applied. Each feature’s one-way ANOVA F-statistic and associated *p*-value was calculated using Python’s sci-kit learn package (sklearn.feature selection.f classif)^64^. The *F*-statistic is the ratio of the variation between group means and the variation within groups. A higher *F*-statistic is associated with greater evidence that there is a difference between group means. Any feature with a *p*-value greater than 0.05 was removed from the feature set. This resulted in pre-filtered static and active feature sets. The pre-filtered static task and active task feature sets were utilized separately and combined to develop machine-learning classifier models. Note that this *p*-value filtering aimed to reduce the feature set for sequential feature selection. The purpose was not to claim the statistical significance of features, as this was not a conservative statistical analysis.

### Sequential feature selection

The pre-filtered feature sets were further reduced by forward sequential feature selection using Python’s sci-kit learn package (sklearn.feature selection.SequentialFeatureSelector)^64^. This process further reduced the feature set and determined an optimal subset of features for each classification model. In forward sequential feature selection, features were sequentially added to the training and testing of a machine-learning model. At each stage, the model chose the best feature to add based on the five-fold cross-validation F1 score of the model. This was repeated until all features had been sequentially added to the model and assessed for the five-fold cross-validation F1 score.

After the forward sequential feature selection was completed, the optimal feature subsets for each model were identified. The optimal feature subset was defined as the subset of features that resulted in the highest five-fold cross-validation F1 score, where the F1 score is the harmonic mean precision and recall of the model’s predictions. Thus, for young controls vs. PD, age-matched controls vs. PD, and PD non-fallers vs. PD fallers, there were five different subsets of static features, five different subsets of active features, and five different subsets of static+active features, one corresponding to each model.

### Model development and tuning

After identifying the optimal feature subsets for each model, the models were further refined through hyperparameter tuning. The previously determined optimal subset for the model was used to tune the model during a five-fold cross-validation. A grid search was used for hyperparameter tuning, with the goal of hyperparameter tuning to reduce the overfitting of the model to the training set of data.

### Model evaluation and comparison

After tuning the hyperparameters for each model, their performance was analyzed using a stratified five-fold cross-validation with optimal feature subsets. For each fold in the cross-validation, the model’s parameters were determined from the training set of data, and the performance of the trained models was determined from the separate test set of data. Since each participant only completed each task once, participants did not overlap between each fold’s training and testing sets. The average accuracy, precision, recall, F1, and kappa scores were computed at each fold and then averaged. The average receiver operating characteristic curve (ROC) curve and area under the curve (AUC) were also computed from the cross-validation. The ROC curve showed the classification model performance at all classification thresholds, and the AUC was the area under this curve. An AUC of one represented a perfect classification model, and an AUC of 0.5 represented a model that classifies at the same level as random chance. Each of these metrics was computed using Python’s sci-kit learn package^64^.

A mixed effects model was conducted with each classifier’s performance metric used as the response variable, while classifier architecture type and feature set task type (static task vs. static+active task) were fixed effects. The same analysis was conducted for active tasks vs. static+active tasks and active tasks vs. static tasks. A significance level of *p* < 0.05 was adjusted using a Bonferroni correction for the three comparisons, resulting in a corrected significance level of *p* < 0.017.

### Misclassification analysis

Individuals who were commonly misclassified across models were identified (Supplementary Figs. 4–7; Supplementary Tables 6 and 7). This process isolated participant-specific postural control strategies or characteristics that contributed to incorrect classifications. Commonly misclassified subjects were defined as individuals who were misclassified in at least three of the five models. This was determined separately for models that used static, active, and static+active data subsets, thus resulting in three sets of commonly misclassified subjects.

### Common feature identification and analysis

Feature analysis was used to focus on specific COP-derived features to investigate altered postural control in PD and in PD fallers. Common features were defined as features that appeared in a subset of at least three of the five models. A *z*-score of each common feature per group and Pearson’s pairwise correlation across common features was computed. The correlation across features is shown in Supplementary Fig. 8. The specific tasks (eyes open, eyes closed, one foot, gait, functional reach, bend over), types of features (positional, dynamic, and frequency), and type of COP measure (average and asymmetric) contributing to the set of common features were identified.

### Shapley value analysis

The Shapley values for each feature contributing to each model were also calculated. The purpose of Shapley analysis is to determine the marginal contribution of each feature used in the machine-learning model, relative to the other features^65^. A Shapley value is defined as the average marginal contribution of a feature value across all possible coalitions. In simple terms, not all features contribute evenly to a model’s final output, and the Shapley value is a way to determine how a feature might have contributed more or less than the others. Python’s SHAP package was used to calculate Shapley values^66^ and develop the visualization plots shown in Supplementary Figs. 9 and 10. The single feature with the highest Shapley value for each model was identified, which resulted in a top Shapley feature set for static, active, and static+active task models.

### Intersection of common and top Shapley value features

Features that appeared in the common feature sets and top Shapley value feature sets were further analyzed for their biomechanical significance. These key features were used to train and test five machine-learning model architectures with each model’s performance metrics of accuracy, precision, recall, F1, and kappa determined by a five-fold cross-validation. Key feature names, distributions, and model performance results are shown in Supplementary Fig. 11.

### Reporting summary

Further information on research design is available in the Nature Research Reporting Summary linked to this article.

### Supplementary information

Supplemental Material

Reporting Summary

## Data Availability

The datasets generated and analyzed in this study are available through our github repository: https://github.com/NRTL-Repository/Herbers2024_NPJ_InsolePressure.

## Code availability

Similarly, code for this study is available through our github repository: https://github.com/NRTL-Repository/Herbers2024_NPJ_InsolePressure.
