# Supplementary material for: Distinguishing features of Parkinson’s disease fallers based on wireless insole plantar pressure monitoring
Source: NPJ Parkinsons Dis. 2024 Mar 19;10:67. doi: 10.1038/s41531-024-00678-2 (PMC10951221; doi:10.1038/s41531-024-00678-2)
Supplement: Supplementary file 1 — Supplemental Material [file 41531_2024_678_MOESM1_ESM.pdf]

Supplementary Data

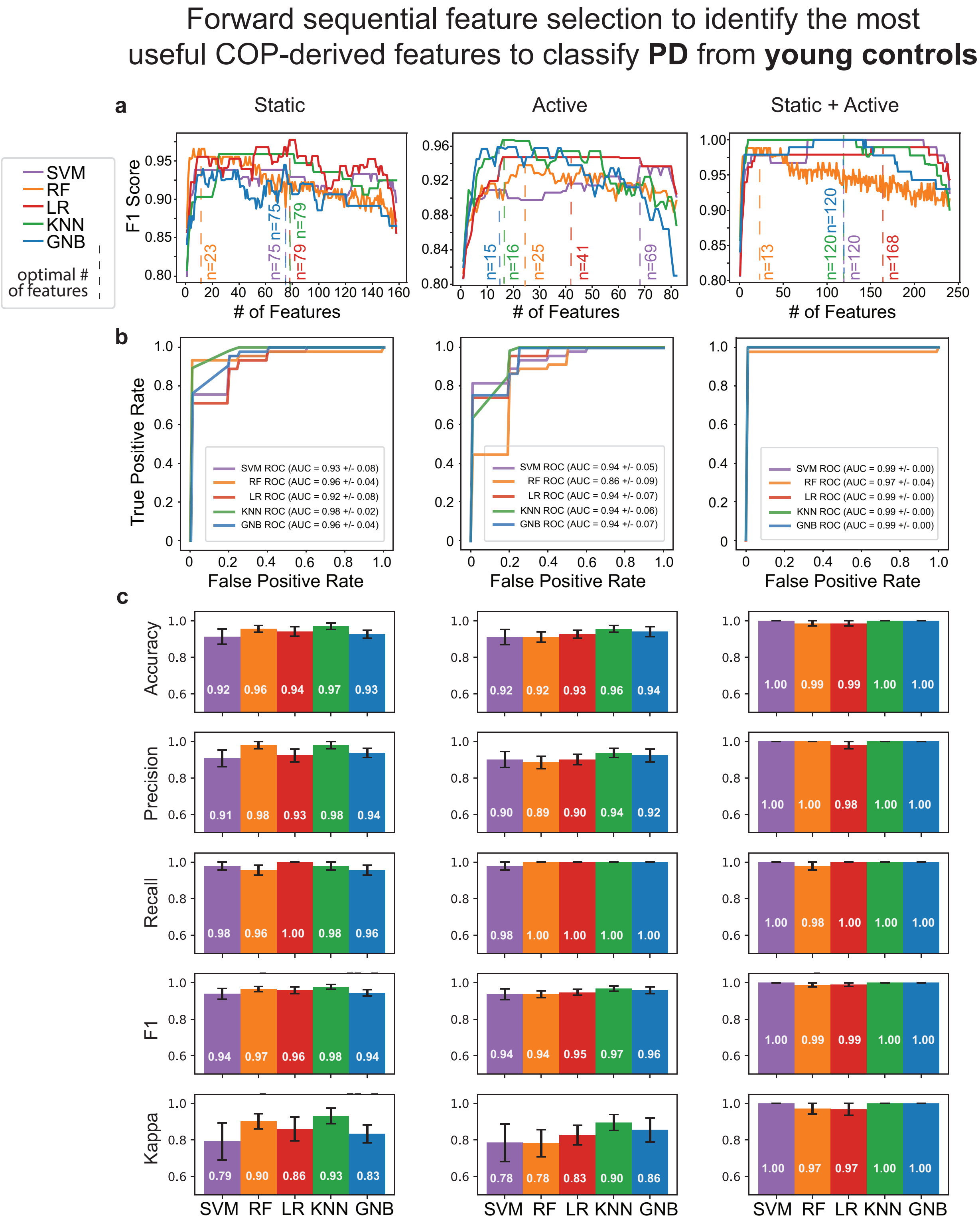

Supplementary Figure 1. Feature selection and model performance analysis between individuals with PD and young control subjects. Forward sequential feature selection was used to determine the optimal subset of features for SVM, RF, LR, KNN, and GNB models. (a) The F1 score was then calculated for each subset of features using a five-fold cross-validation. The optimal subset of features for each model was determined by the maximum F1 score (denoted by a dashed line and n value, where n is the number of features in the optimal subset). Hyperparameters were tuned using the optimal subset of features for each model. Five-fold cross-validation of the tuned model was used to determine (b) the ROC curve and AUC metrics, and (c) the resulting performance metrics of accuracy, precision, recall, F1, and kappa.

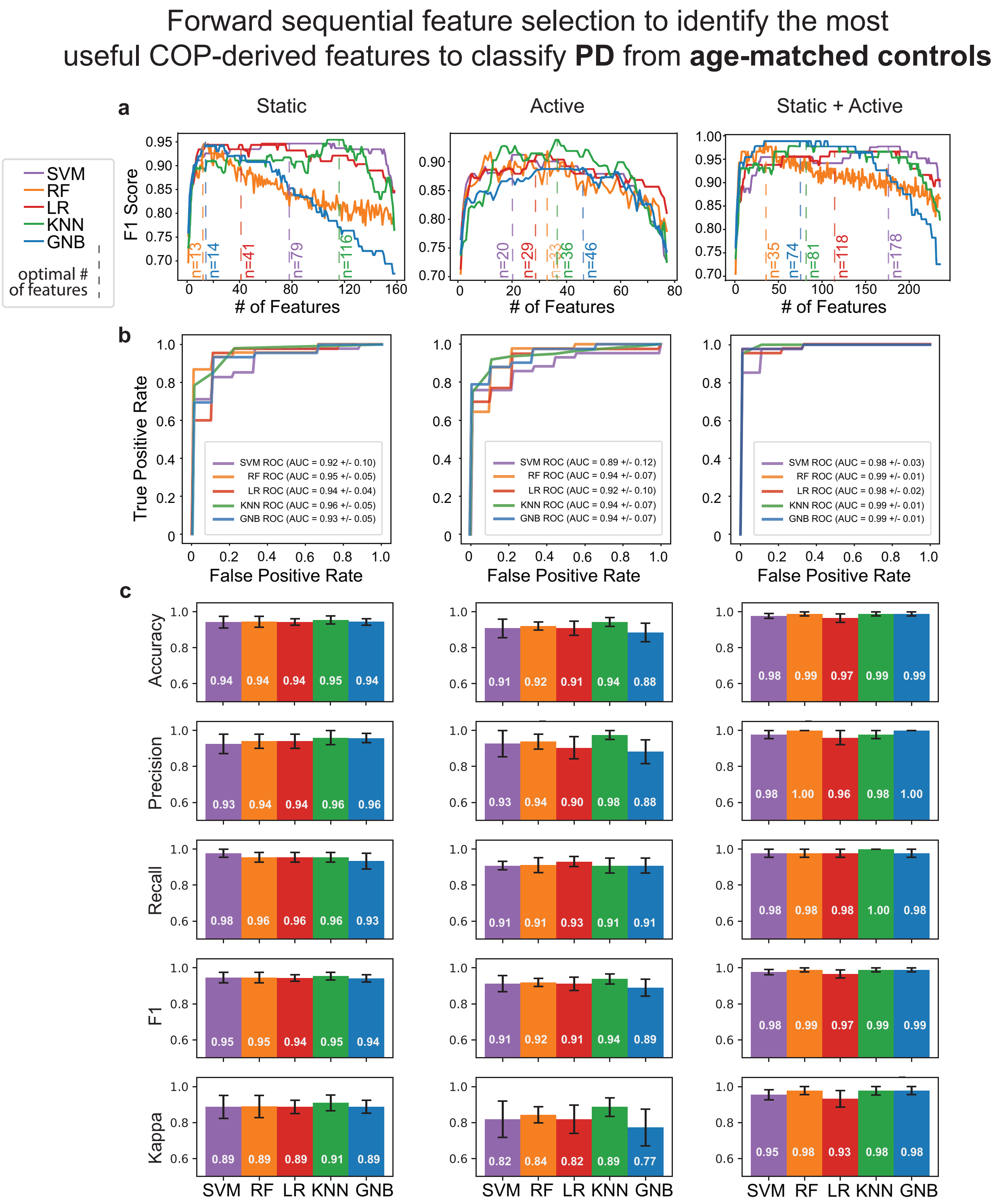

Supplementary Figure 2. Feature selection and model performance analysis between individuals with PD and age-matched control subjects. (a-c) follow the description given in the caption of Fig. S1.

Forward sequential feature selection to identify the most useful COP-derived features to classify **PD fallers** from **PD non-fallers**

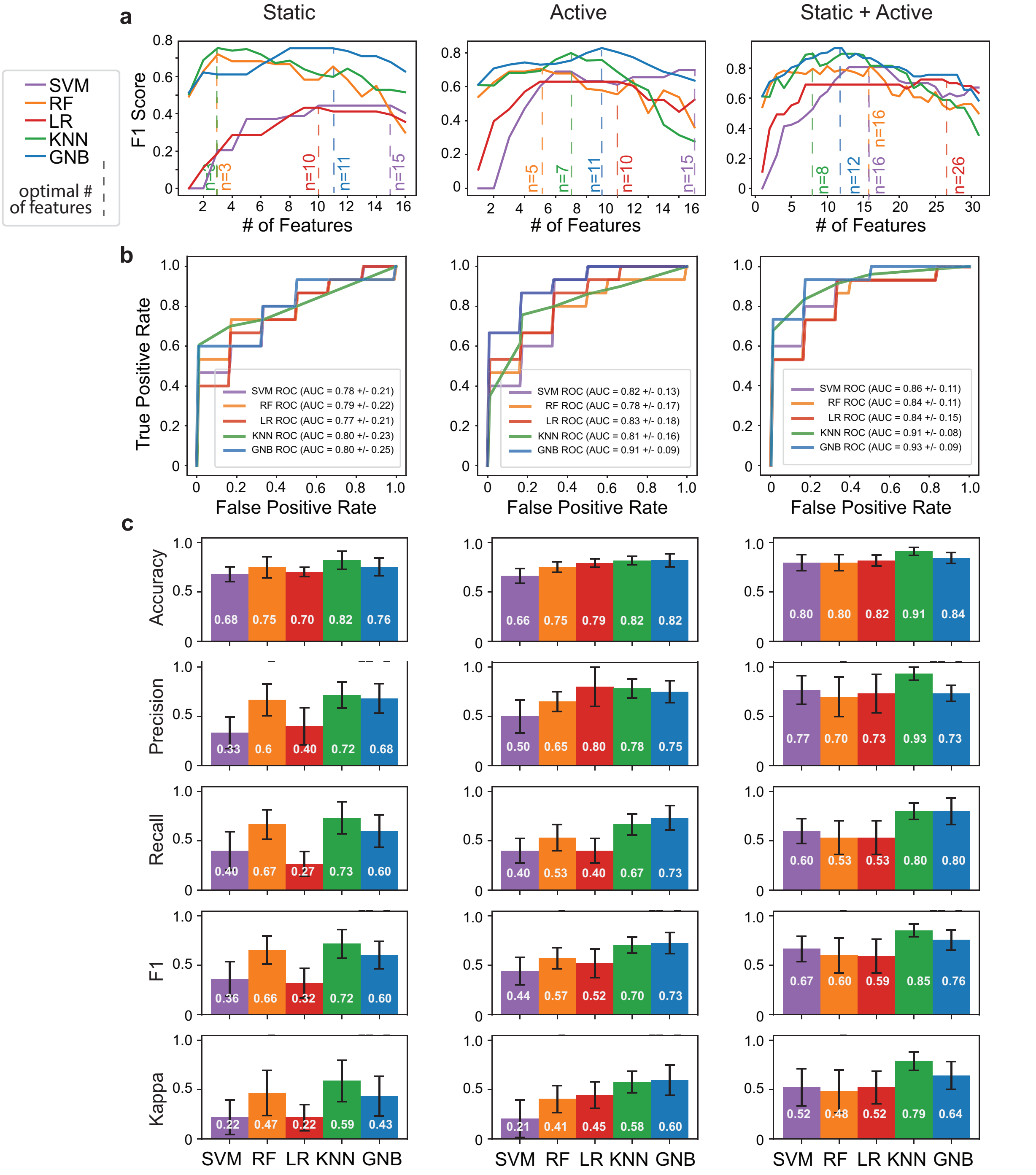

Supplementary Figure 3. Feature selection and model performance analysis between PD faller and PD non-faller groups. (a-c) follow the description given in the caption of Fig. S1.

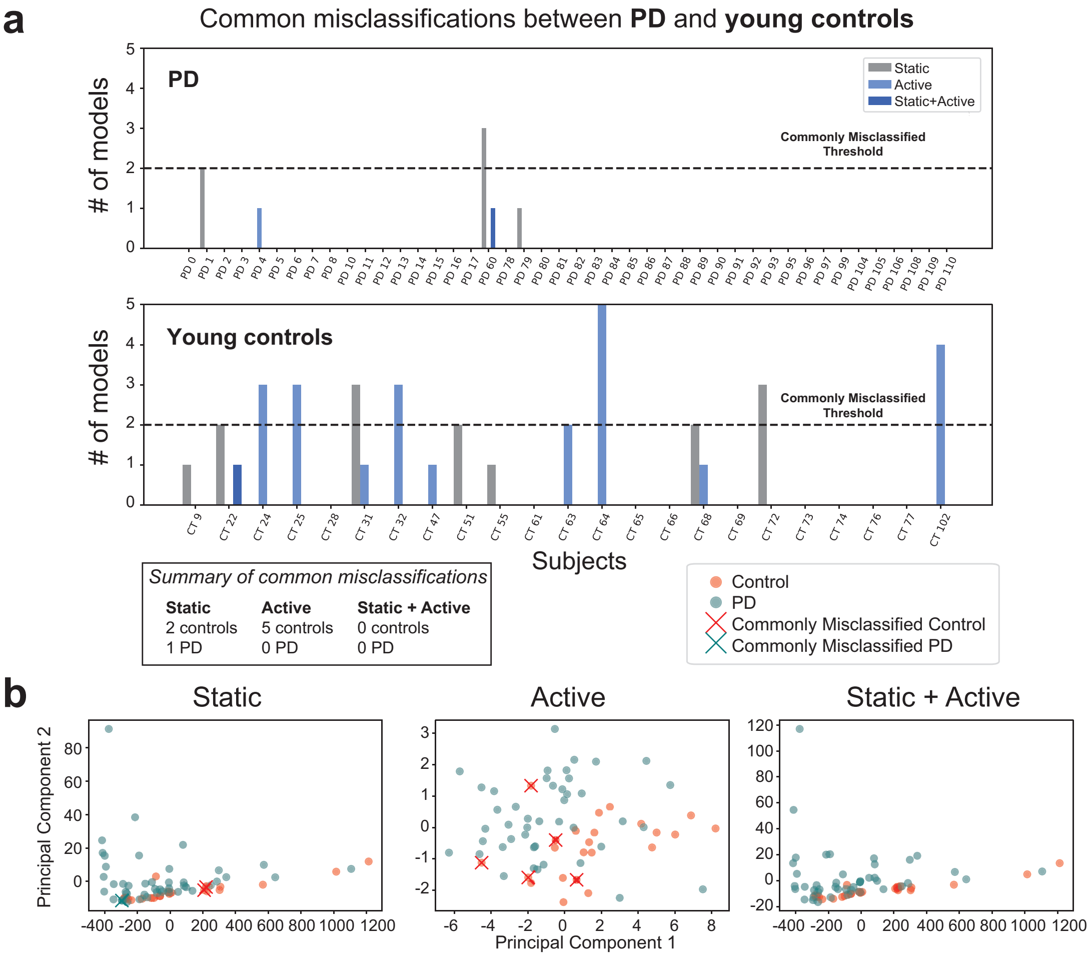

Supplementary Figure 4. Misclassification analysis for PD and young control classifiers. (a) The number of times each subject was misclassified across all five models for static, active, and static+active task feature sets. Subjects who were commonly misclassified (misclassified 3 times or more) surpass the shown misclassification threshold. (b) The first and second principal components of common features for each task feature set are shown, with commonly misclassified subjects within that feature set marked with an ’X’.

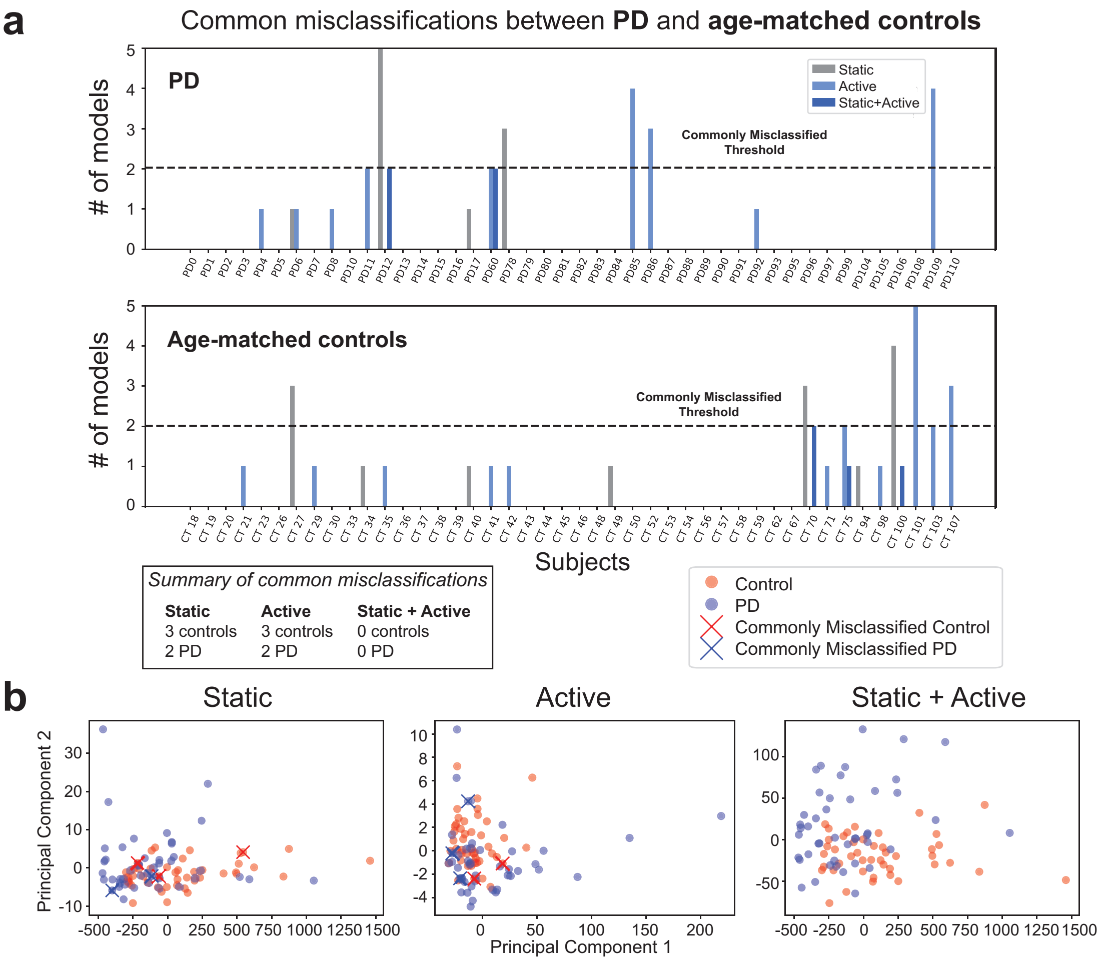

Supplementary Figure 5. Misclassification analysis for PD and age-matched control classifiers. (a-b) follow the description given in caption Fig. S4. It is likely that these misclassified individuals represent edge-cases of postural control, relative to their groups, which could not consistently be learned by the models. A visualization of these edge-case characteristics can be seen in the principal component analysis (PCA) representation that highlights individuals who were and were not commonly misclassified. The commonly misclassified individuals were typically close to or overlap the clusters of the two groups. Note that the PCA plots are a two-dimensional representation of much higher dimensional feature space, and therefore are an incomplete representation of the separation between classes. Expanding the data set by including additional balance tasks (like reactive tasks) may be useful to reduce the misclassification of edge-cases and outliers. Also, notably, there was a lack of redundancy between commonly misclassified subjects for models trained on static, active, and static+active task features. Commonly misclassified subjects were eliminated when both static and active task features were available for the model. Static task and active task features likely covered different dimensionalities of the postural control feature space, and thus improved the separation hyperplane between groups that was learned by the model. This finding thus re-iterates the clinical need for quantification of a variety of balance assessments to fully capture the high dimensionality of postural instability. Age, sex, and surface type of commonly misclassified PD vs. age-matched controls can be seen in **Supplementary Table 6**. There was no apparent theme as to whether demographic factors (age, sex) contributed to these misclassifications. However, many of the individuals who were misclassified (4/5) had data collected at the same location, and all individuals (5/5) completed the tasks on the same type of surface (hardwood floor).

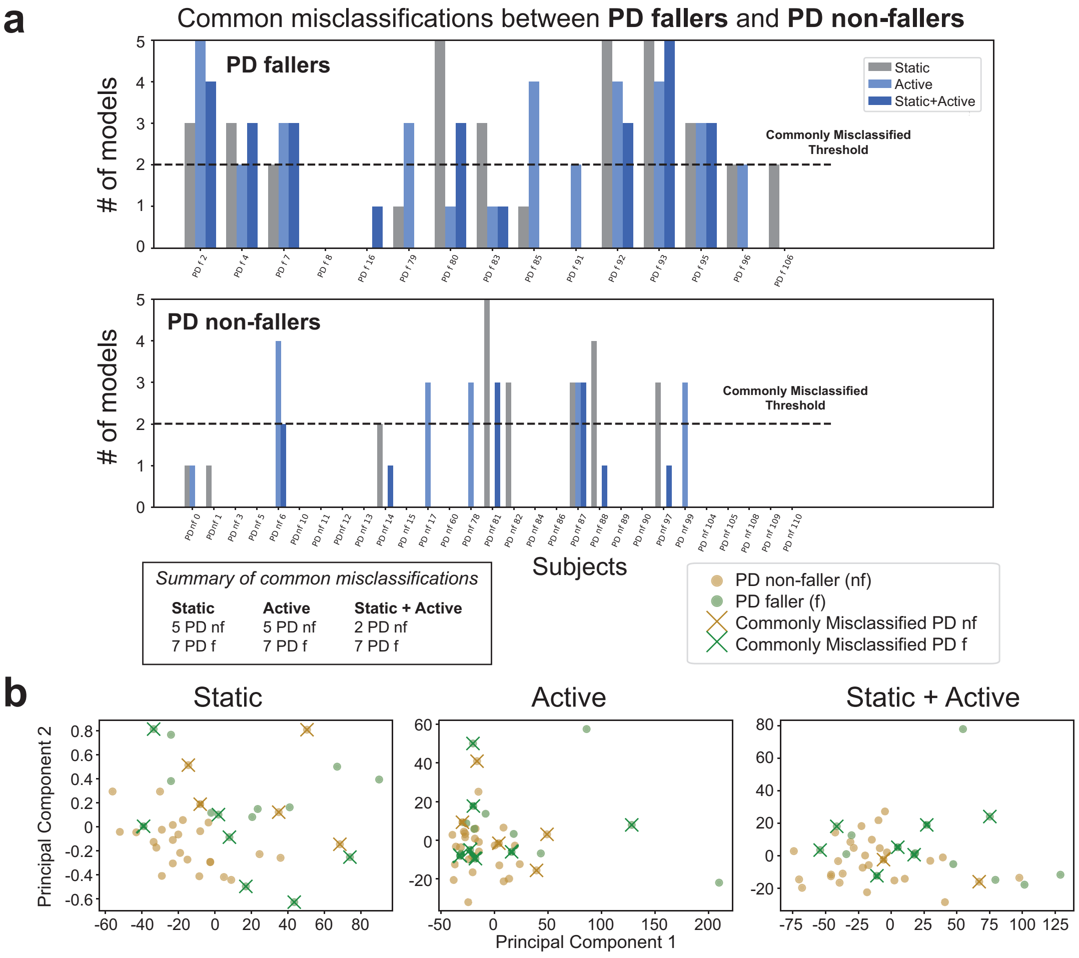

Supplementary Figure 6. Misclassification analysis for PD faller and PD non-faller classifiers. (a-b) follow the description given in caption Fig. S4. Additionally, there are a few other likely reasons which may contribute to these misclassifications. (1) The dataset was imbalanced, with 15 in the PD fallers group and 29 in the PD non-fallers group. Falls in PD occur for a handful of underlying reasons, such as poor proprioception, an altered perception of verticality, bradykinesia, or a combination of these reasons. The underlying COP characteristics that represent a PD faller vary across participants and tasks. Due to the sample size, the model may not have been able to learn all edge-cases of the COP-derived features to robustly classify PD fallers. If a larger and broader group of PD fallers was used for model training and testing, model classification performance and its ability to identify PD fallers would likely improve. Also, (2) participants self-reported their balance issues and fall history. This method of reporting introduces subjectivity to the class labels, as balance issues and fall history may be interpreted differently across the participants. There may also be environmental components of fall history that were outside the ability of the questionnaire to probe (for example, someone who is a faller may fall due to their hobby of riding a unicycle versus someone who does not). Utilizing a clinician or trained balance examiner to identify individuals with balance issues and fall history would decrease the subjectivity of determining these class labels. Age, sex, and surface type of commonly misclassified PD fallers vs PD non-fallers can be seen in **Supplementary Table 7**.

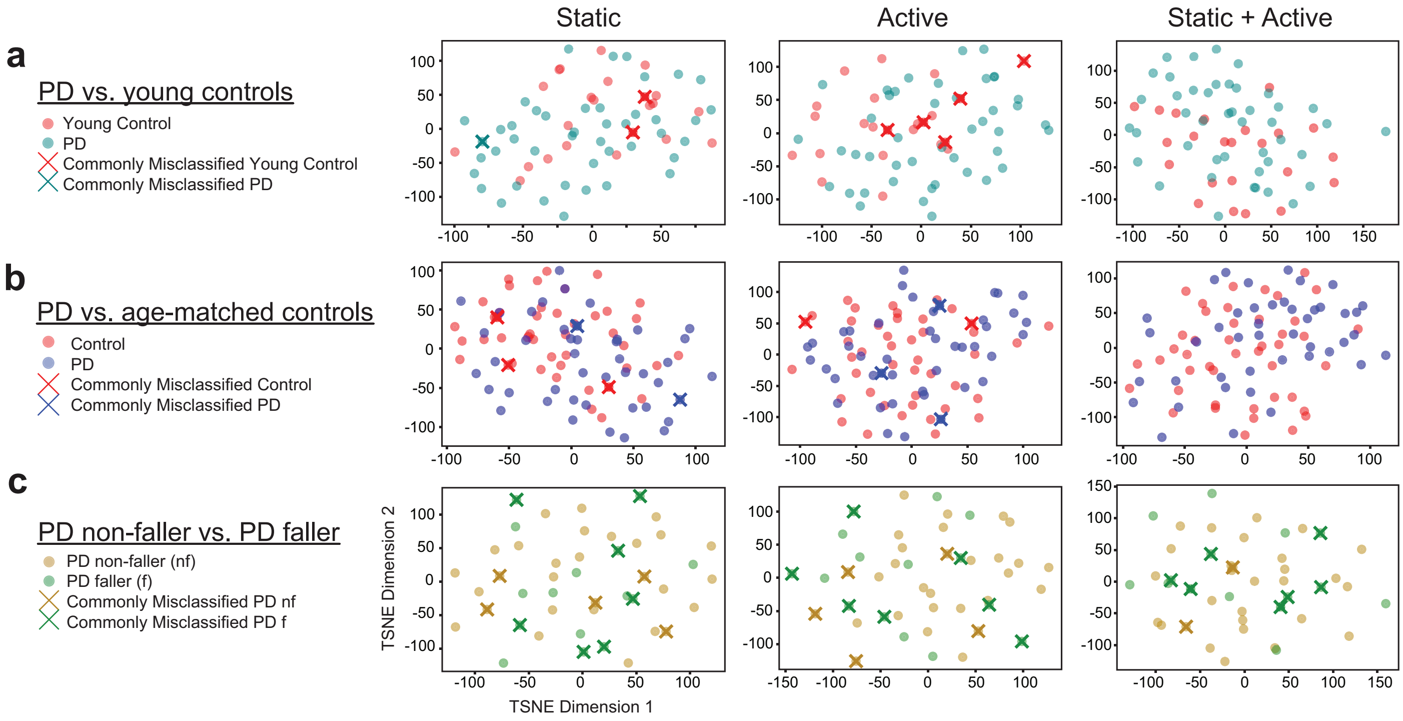

Supplementary Figure 7. Additional visualization of the misclassification analysis for (a) PD and young controls, (b) PD and age-matched controls, and (c) PD faller and PD non-faller classifiers was completed using t-distributed Stochastic Neighbor Embedding (TSNE). Similar to principal component analysis, TSNE is a dimensionality reduction that is well-suited to visualizing high-dimensional data. However, unlike principal component, TSNE considers non-linear local and global geometric structures in its transformation. The first and second TSNE dimensions of common features for each task feature set are shown, with commonly misclassified subjects within that feature set marked with an 'X'. The visualization of commonly misclassified subjects using TSNE plots, did not clearly demonstrate class separability, or highlight any interesting characteristics of commonly misclassified individuals. This is likely because TSNE is a way to visualize classes based on distance from each other, and while these classes were able to be differentiated via machine learning models demonstrated in this paper, the Euclidean distance between classes is likely not the main mode of separation, and more likely due to some linear or non-linear separation. Additionally, these TSNE plots show a two-dimensional representation of a higher dimensional feature space, resulting in an incomplete visualization of the separation between the higher dimensional classes.

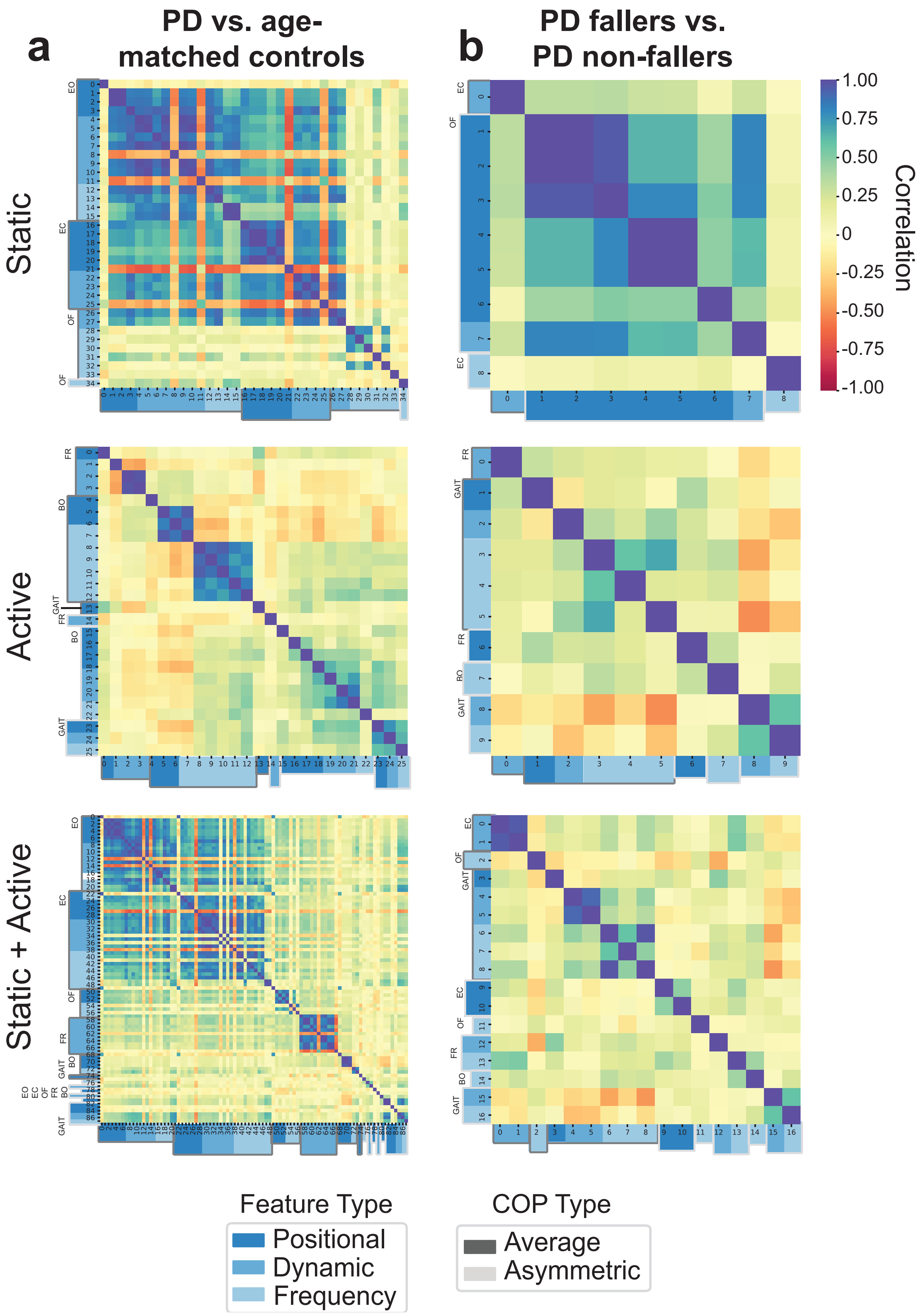

Supplementary Figure 8. Common features, chosen by at least three of the five models during feature selection, for (a) PD vs. age-matched controls and (b) PD faller vs. PD non-faller classifiers. Common features were identified for models trained on static task features only, active task features only, and static+active task features. Correlation across features is shown. The surrounding bars indicate each feature's domain. The outline of the bars shows whether the feature was related to the average or asymmetry across feet. Corresponding names and details of features can be seen in **Supplementary Tables 8-9**.

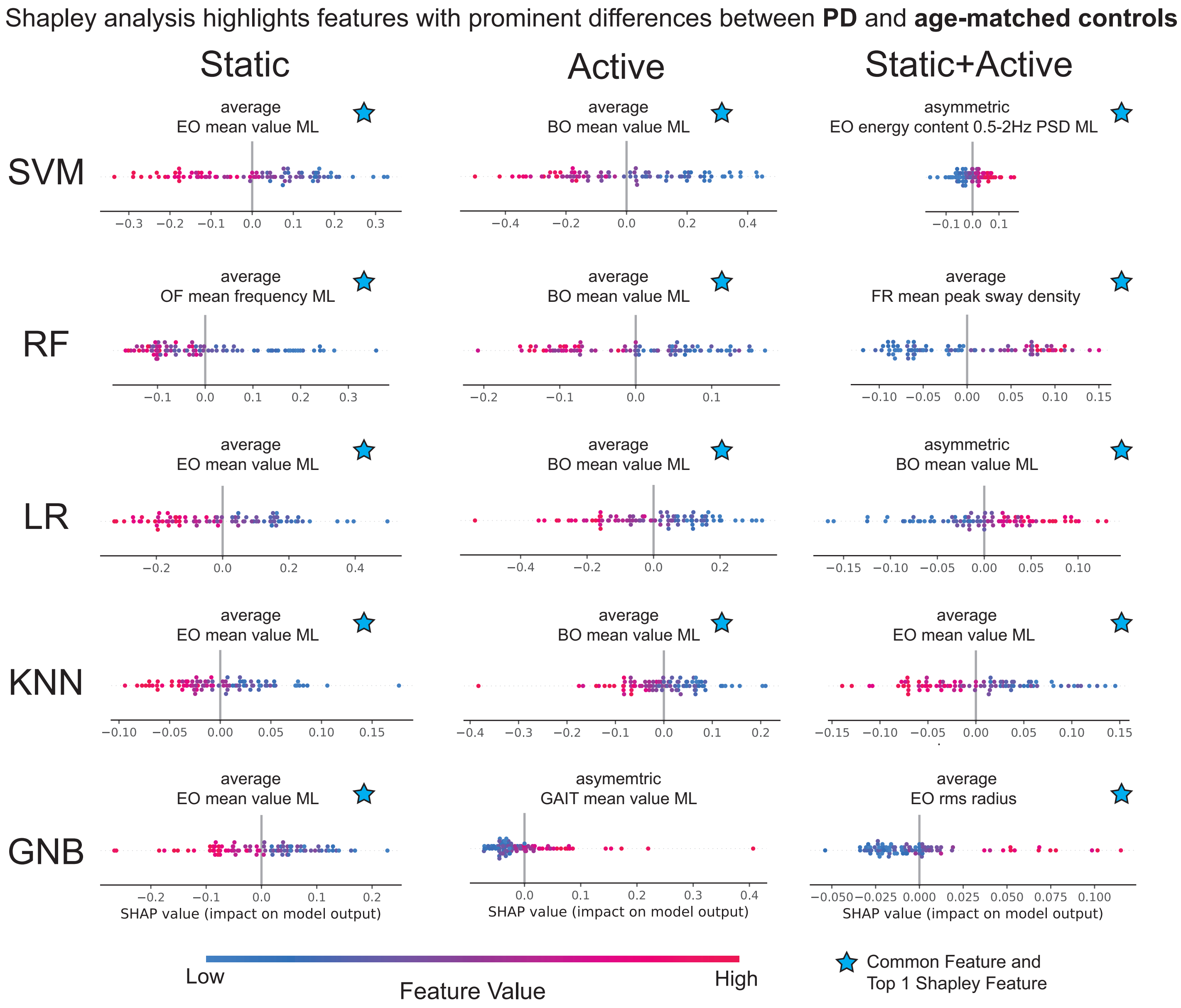

Supplementary Figure 9. The top feature based on Shapley value for each feature set and each model classifying PD from age-matched control subjects. Features that also appeared as a common feature are marked with a blue star.

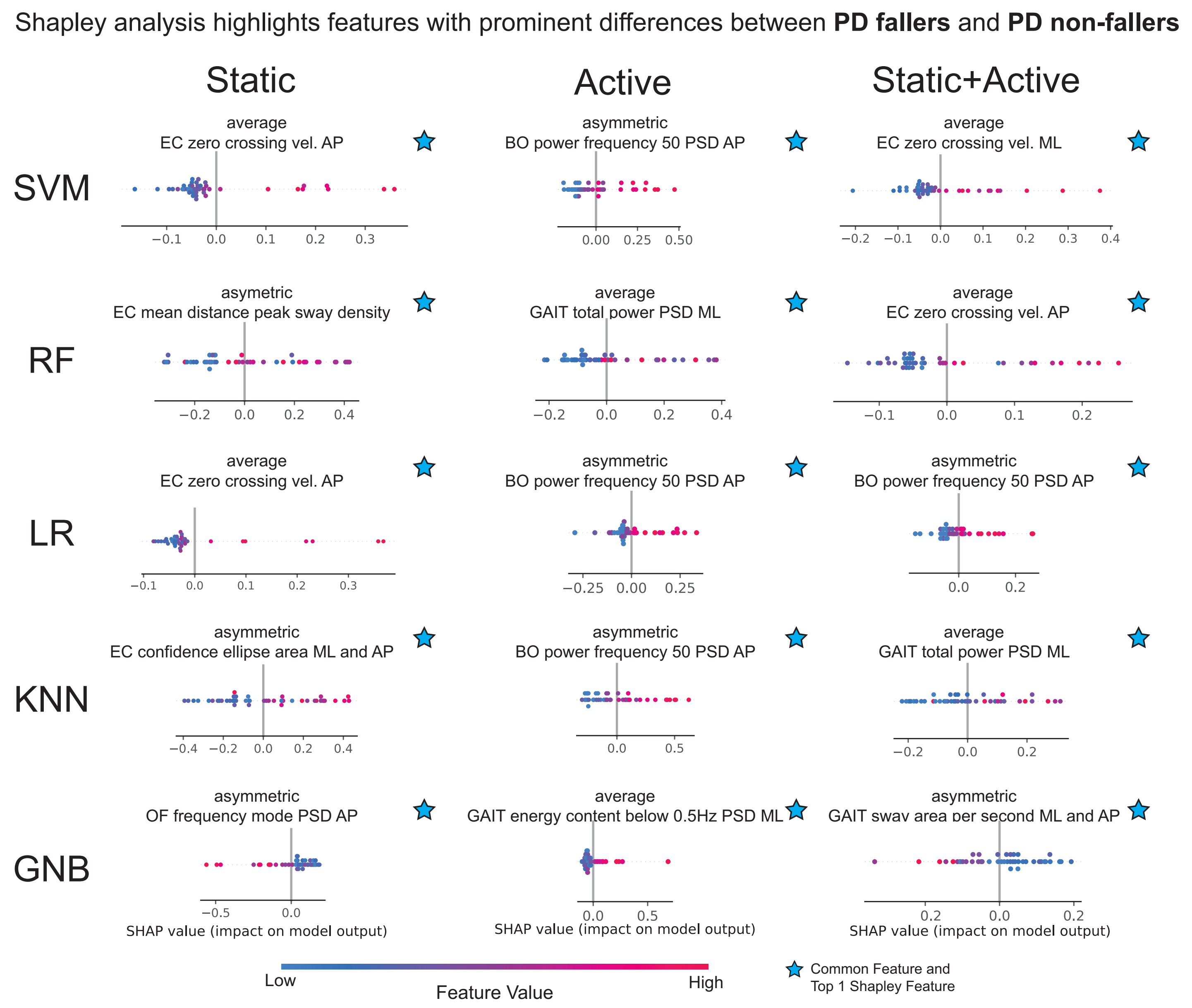

Supplementary Figure 10. The top feature based on Shapley value for each feature set and each model classifying PD fallers from PD non-fallers. Features that also appeared as a common feature are marked with a blue star.

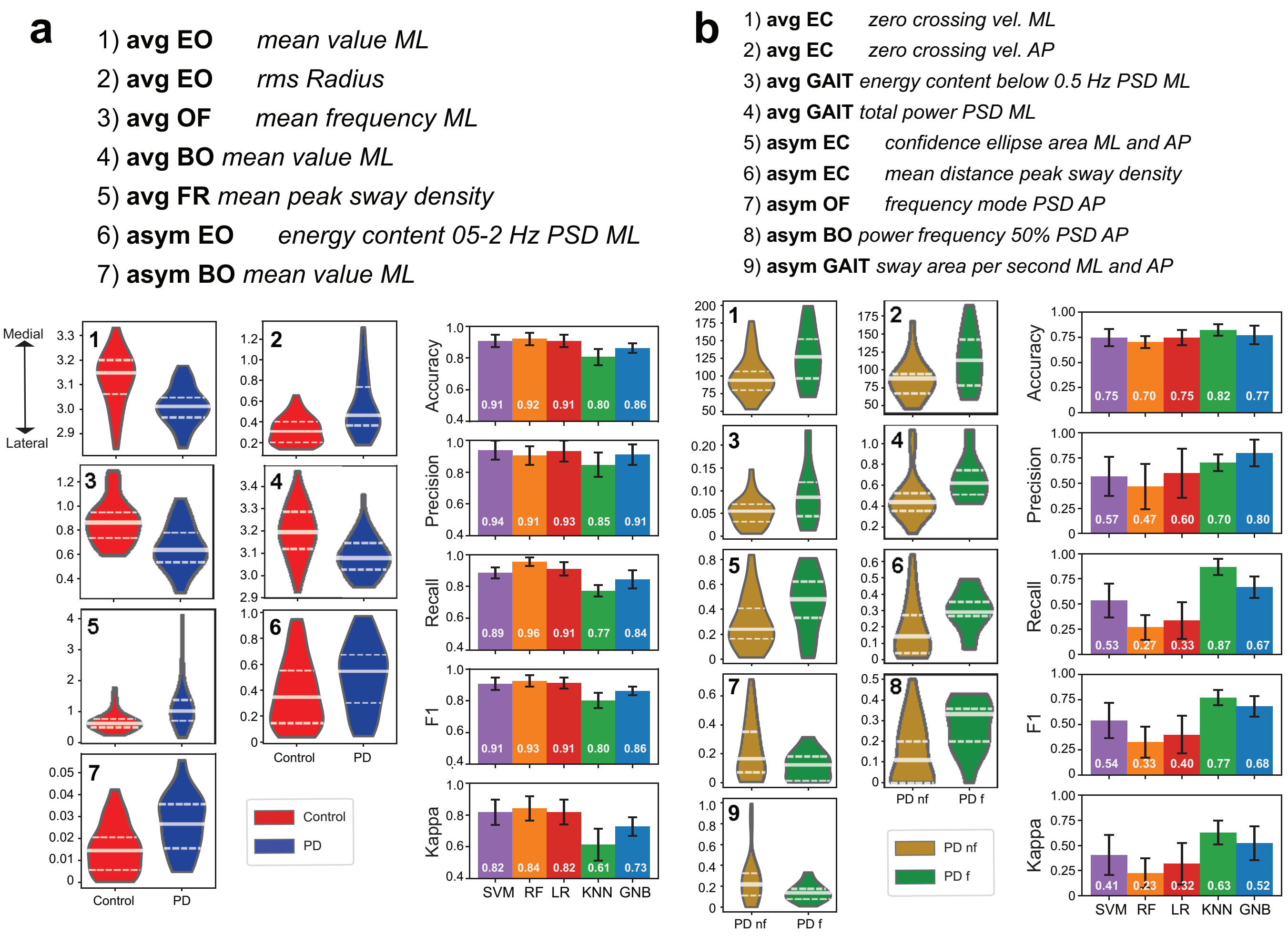

Supplementary Figure 11. The top Shapley and common features were identified and analyzed for (a) PD vs. age-matched controls and (b) PD fallers vs. PD non-fallers. The distribution of features between groups are shown. The mean of each group is marked by a solid line, and the quartiles of the distribution are marked by dashed lines. These features were used to train and test five different model architectures. The average performance metrics (with standard error mean) from a five-fold cross-validation for each model are shown.

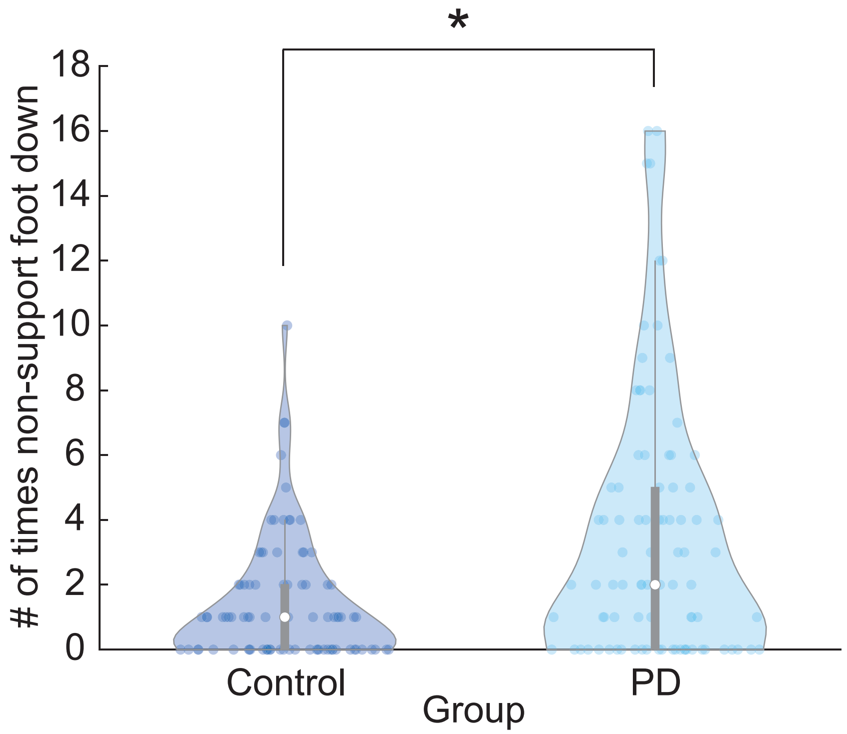

Supplementary Figure 12. The number of times an individual placed their foot down during the one foot stance for age-matched controls and individuals with PD. Comparison of the two groups was significant (Wilcoxon rank-sum test, two-sided, $p<0.001$).

Supplementary Table 1: Locations and support surfaces of data collection. A significant difference in surface types was observed between PD and age matched controls (p<0.001), and between PD and young controls (p<0.001).

Participant	Location	Surface	Participant	Location	Surface
1	Participant's home	Hardwood	57	State Fair	Concrete
2	Participant's home	Hardwood	58	State Fair	Concrete
3	Participant's home	Hardwood	59	State Fair	Concrete
4	Local park	Pavement	60	State Fair	Concrete
5	Local park	Pavement	61	State Fair	Concrete
6	Local park	Pavement	62	State Fair	Concrete
7	Local park	Pavement	63	State Fair	Concrete
8	Local park	Pavement	64	State Fair	Concrete
9	Local park	Pavement	65	State Fair	Concrete
10	Local park	Pavement	66	State Fair	Concrete
11	Local park	Pavement	67	State Fair	Concrete
12	Local park	Pavement	68	State Fair	Concrete
13	Local park	Pavement	69	State Fair	Concrete
14	Participant's home	Hardwood	70	State Fair	Concrete
15	Participant's home	Hardwood	71	State Fair	Concrete
16	Participant's home	Hardwood	72	State Fair	Concrete
17	Participant's home	Hardwood	73	State Fair	Concrete
18	Participant's home	Hardwood	74	State Fair	Concrete
19	State Fair	Concrete	75	State Fair	Concrete
20	State Fair	Concrete	76	State Fair	Concrete
21	State Fair	Concrete	77	State Fair	Concrete
22	State Fair	Concrete	78	State Fair	Concrete
23	State Fair	Concrete	79	Participant's home	Hardwood
24	State Fair	Concrete	80	Participant's home	Hardwood
25	State Fair	Concrete	81	Participant's home	Hardwood
26	State Fair	Concrete	82	Participant's home	Hardwood
27	State Fair	Concrete	83	Participant's home	Hardwood
28	State Fair	Concrete	84	Local gym	Hardwood
29	State Fair	Concrete	85	Local gym	Hardwood
30	State Fair	Concrete	86	Local gym	Hardwood
31	State Fair	Concrete	87	Local gym	Hardwood
32	State Fair	Concrete	88	Local gym	Hardwood
33	State Fair	Concrete	89	Local gym	Hardwood
34	State Fair	Concrete	90	Local gym	Hardwood
35	State Fair	Concrete	91	Local gym	Hardwood
36	State Fair	Concrete	92	Local gym	Hardwood
37	State Fair	Concrete	93	Local gym	Hardwood
38	State Fair	Concrete	94	Local gym	Hardwood
39	State Fair	Concrete	95	Local gym	Hardwood
40	State Fair	Concrete	96	Local gym	Hardwood
41	State Fair	Concrete	97	Local gym	Hardwood
42	State Fair	Concrete	98	Local gym	Hardwood
43	State Fair	Concrete	99	Participant's home	Hardwood
44	State Fair	Concrete	100	Participant's home	Hardwood
45	State Fair	Concrete	101	Local gym	Turf
46	State Fair	Concrete	102	Local gym	Turf
47	State Fair	Concrete	103	Local gym	Turf
48	State Fair	Concrete	104	Local gym	Turf
49	State Fair	Concrete	105	Local gym	Hardwood
50	State Fair	Concrete	106	Local gym	Hardwood
51	State Fair	Concrete	107	Local gym	Hardwood
52	State Fair	Concrete	108	Local gym	Hardwood
53	State Fair	Concrete	109	Local church	Carpet
54	State Fair	Concrete	110	Local church	Carpet
55	State Fair	Concrete	111	Local church	Carpet
56	State Fair	Concrete			

Supplementary Table 2: Variables used in feature equations.

Variable Name	Definition
T	Total duration of the signal
N	Number of points in the signal
F_s	Sampling frequency
ML_n	ML coordinates
AP_n	AP coordinates
X_n	Centered ML coordinates
Y_n	Centered AP coordinates
R_n	Radius
$COV\ AP$	Covariance AP
SD_n	Sway density
z_l	Zero crossing of velocity
p_l	Velocity peaks
V_n^x	ML velocity
V_n^y	AP velocity
V_n	Velocity norm
Γ_k^s	Power spectral density (PSD) of S for frequency kF_s/N
k_{inf}	PSD bounds: $k_{inf} = \left[0.15\frac{N}{F_s}\right]$
k_{sup}	PSD bounds: $k_{sup} = \left[5\frac{N}{F_s}\right]$
M_l^S	l-th spectral moment of S

Supplementary Table 3: Positional features from per-foot COP throughout the duration of each task.

Name	Definition	Equation	
Mean ML	Mean position of the COP trajectory for ML coordinates	$\frac{1}{N}\sum_n ML_n$	(P1)
Mean AP	Mean position of the COP trajectory for AP coordinates	$\frac{1}{N}\sum_n AP_n$	(P2)
Mean Distance ML	Mean distance of the COP from the center of the trajectory for ML coordinates	$\frac{1}{N}\sum_n \lvert X_n \rvert$	(P3)
Mean Distance AP	Mean distance of the COP from the center of the trajectory for AP coordinates	$\frac{1}{N}\sum_n \lvert Y_n \rvert$	(P4)
Mean Distance	Mean Euclidean distance of the COP from the center of the trajectory	$\frac{1}{N}\sum_n \lvert R_n \rvert$	(P5)
Maximal Distance ML	Maximal distance of the COP from the centroid for ML coordinates	$max_n \lvert X_n \rvert$	(P6)
Maximal Distance AP	Maximal distance of the COP from the centroid for AP coordinates	$max_n \lvert Y_n \rvert$	(P7)
Maximal Distance Radius	Maximal Euclidean distance of the COP from the centroid	$max_n \lvert R_n \rvert$	(P8)
RMS ML	Root mean square of centered trajectory for ML coordinates	$\sqrt{\frac{1}{N}\sum_n {X_n}^2}$	(P9)
RMS AP	Root mean square of centered trajectory for AP coordinates	$\sqrt{\frac{1}{N}\sum_n {Y_n}^2}$	(P10)
RMS Radius	Root mean square of centered trajectory	$\sqrt{\frac{1}{N}\sum_n {R_n}^2}$	(P11)
Range ML	Range of COP path for ML coordinates	$max_{n,m} \lvert X_n - X_m \rvert$	(P12)
Range AP	Range of COP path for AP coordinates	$max_{n,m} \lvert Y_n - Y_m \rvert$	(P13)
Range	Range of COP path	$max_{1 \le n \le m < N}\left(\sqrt{(X_n - X_m)^2 + (Y_n - Y_m)^2}\right)$	(P14)
Range Ratio	Ratio of ML and AP ranges	$\frac{Range\ ML}{Range\ AP}$	(P15)
Planar Deviation	Square root of the sum of the variances of displacements in ML and AP directions	$\sqrt{RMS\ ML^2 + RMS\ AP^2}$	(P16)

Coefficient of Sway Direction	Coefficient of correlation between the ML and AP trajectories	$\frac{COV}{RMS\ ML\ \times\ RMS\ AP}$	(P17)
95% Confidence of Sway Direction	Area of the ellipse that contains the true mean of $(X_n, Y_n)_{\leq\leq N}$ with a probability of 95%	See Definition	(P18)
Principal Sway Direction	First PCA component of COP that identifies the direction of maximum dispersion of the COP trajectory	$arccos\left(\frac{\lvert v_2\rvert}{\sqrt{v_1^2 + v_2^2}}\right)$	(P19)

Supplementary Table 4: Dynamic features from per-foot COP throughout the duration of each task. Features marked by * were included in the open source code provided by Quijoux et al.[54], but not included in the feature set generation for the work presented in this paper.

Name	Definition	Equation	
*Sway Length ML	Sway length for ML coordinates	$\sum_n \lvert X_{n+1} - X_n\rvert$	(D01)
*Sway Length AP	Sway length for AP coordinates	$\sum_n \lvert X_{n+1} - X_n\rvert$	(D02)
*Sway Length	Total sway length	$\sum_n \lvert X_{n+1} - X_n\rvert$	(D03)
Mean Velocity ML	Mean velocity of the COP for ML coordinates	$\frac{Sway\ Length\ ML}{T}$	(D04)
Mean Velocity AP	Mean velocity of the COP for AP coordinates	$\frac{Sway\ Length\ AP}{T}$	(D05)
Mean Velocity	Mean velocity of the COP	$\frac{Sway\ Length}{T}$	(D06)
Sway Area per Second	Average area circumscribed by the COP for each 1 second time interval	$\frac{1}{2T}\sum_n \lvert X_{n+1}\, Y_n - X_n\, Y_{n+1}\rvert$	(D07)
*Standard Deviation Velocity ML	Deviation of the velocity of the COP for ML coordinates	$\sqrt{\frac{1}{N}\sum_n (V_n^x - \overline{V^x})^2}$	(D08)
*Standard Deviation Velocity AP	Deviation of the velocity of the COP for AP coordinates	$\sqrt{\frac{1}{N}\sum_n (V_n^y - \overline{V^y})^2}$	(D09)
*Standard Deviation Velocity	Deviation of the velocity of the COP	$\sqrt{\frac{1}{N}\sum_n (V_n - \bar{V})^2}$	(D10)
Phase Plane Parameter ML	Dispersion of both velocity and position of the COP for ML coordinates	$\sqrt{RMS\ ML^2 + STD\ V^{X^2}}$	(D11)
Phase Plane Parameter AP	Dispersion of both velocity and position of the COP for AP coordinates	$\sqrt{RMS\ AP^2 + STD\ V^{Y^2}}$	(D12)
Length over Area	Total length of the sway path over the surface of the circumscribing area	$\frac{Sway\ Length}{95\%\ Conf\ Area}$	(D13)
Fractal Dimension	Unitless measure of the degree to which a curve fills the space it embeds	See Definition	(D14)
# of Zero Crossings ML	Number of times the COP velocity crosses the zero value axis for ML coordinates	$\#\ Z^{V^x}$	(D15)
# of Zero Crossings AP	Number of times the COP velocity crosses the zero value axis for AP coordinates	$\#\ Z^{V^y}$	(D16)
Mean Positive Peak of ML Velocity	Average of the maximal (+) values between two zero crossings for ML coordinates	See Definition	(D17)
Mean Positive Peak of AP Velocity	Average of the maximal (+) values between two zero crossings for AP coordinates	See Definition	(D18)
Mean Negative Peak of ML Velocity	Average of the maximal (-) values between two zero crossings for ML coordinates	See Definition	(D19)
Mean Negative Peak of AP Velocity	Average of the maximal (-) values between two zero crossings for ML coordinates	See Definition	(D20)

Mean Peak of ML Velocity	Average of the maximal absolute values between two zero crossing for ML coordinates	$\frac{1}{K}\sum_{l} p_l^{V^x}$	(D21)
Mean Peak of AP Velocity	Average of the maximal absolute values between two zero crossing for AP coordinates	$\frac{1}{K}\sum_{l} p_l^{V^y}$	(D22)
Mean Peak Sway Density	Average number of consecutive samples that fall within a circle of given radius of the sway density	$\frac{1}{K}\sum_{l} p_l^{SD}$	(D23)
Mean Spatial Distance between Sway Density Peaks	Average distance between two consecutive peaks in sway density	See Definition	(D24)
Mean Frequency ML	Average frequency of the COP signal for ML coordinates	$\frac{1}{4\sqrt{2}}\left(\frac{MEAN\ V^X}{MEAN\ DIST\ ML}\right)$	(D25)
Mean Frequency AP	Average frequency of the COP signal for AP coordinates	$\frac{1}{4\sqrt{2}}\left(\frac{MEAN\ V^Y}{MEAN\ DIST\ AP}\right)$	(D26)
Mean Frequency ML-AP	Average frequency of the COP signal	$\frac{1}{4\sqrt{2}}\left(\frac{MEAN\ V}{MEAN\ DIST}\right)$	(D27)

Supplementary Table 5: Frequency features from per-foot COP throughout the duration of each task.

Name	Definition	Equation	
Total Power ML	Energy contained in the entire PSD for ML coordinates	$\sum_{k=k_{\infty}}^{k_{sup}} \Gamma_k^X$	(F01)
Total Power AP	Energy contained in the entire PSD for AP coordinates	$\sum_{k=k_{\infty}}^{k_{sup}} \Gamma_k^Y$	(F02)
50% Power Frequency ML	Frequency containing 50% of the PSD for ML coordinates	See Definition	(F03)
50% Power Frequency AP	Frequency containing 50% of the PSD for AP coordinates	See Definition	(F04)
95% Power Frequency ML	Frequency containing 95% of the PSD for ML coordinates	See Definition	(F05)
95% Power Frequency AP	Frequency containing 95% of the PSD for AP coordinates	See Definition	(F06)
Power Mode ML	Dominant frequency in the PSD for ML coordinates	$\frac{F_s}{N} \times \arg max_{k_{inf} \le k \le k_{sup}} \Gamma_k^X$	(F07)
Power Mode AP	Dominant frequency in the PSD for AP coordinates	$\frac{F_s}{N} \times \arg max_{k_{inf} \le k \le k_{sup}} \Gamma_k^Y$	(F08)
Centroidal Frequency ML	Spectral mass location for ML coordinates	$\sqrt{\frac{M_2^X}{M_0^X}}$	(F09)
Centroidal Frequency AP	Spectral mass location for AP coordinates	$\sqrt{\frac{M_2^Y}{M_0^Y}}$	(F10)
Frequency Dispersion ML	Dispersion of both velocity and position of the COP for ML coordinates	$\sqrt{1-\frac{(M_1^X)^2}{M_2^X M_0^X}}$	(F11)
Frequency Dispersion AP	Dispersion of both velocity and position of the COP for AP coordinates	$\sqrt{1-\frac{(M_1^Y)^2}{M_2^Y M_0^Y}}$	(F12)
Energy ≤ 0.5 Hz ML	Energy content at or below 0.5 Hz for ML coordinates	$\sum_{f_{inf} \le f_k \le 0.5} \Gamma^X(f_k)$	(F13)
Energy ≤ 0.5 Hz AP	Energy content at or below 0.5 Hz for AP coordinates	$\sum_{f_{inf} \le f_k \le 0.5} \Gamma^Y(f_k)$	(F14)

Energy 0.5-2 Hz ML	Energy content between 0.5 Hz and 2 Hz for ML coordinates	$\sum_{0.5\le f_k\le 2} \Gamma^X(f_k)$	(F15)
Energy 0.5-2 Hz AP	Energy content between 0.5 Hz and 2 Hz for AP coordinates	$\sum_{0.5\le f_k\le 2} \Gamma^Y(f_k)$	(F16)
Energy > 2 Hz ML	Energy content above 2 Hz for ML coordinates	$\sum_{2< f_k\le f_{sup}} \Gamma^X(f_k)$	(F17)
Energy > 2 Hz AP	Energy content above 2 Hz for AP coordinates	$\sum_{2< f_k\le f_{sup}} \Gamma^Y(f_k)$	(F18)
Frequency quotient ML	Ratio of energy content between 2-5 Hz and energy content at or below 2 Hz for ML coordinates	$\frac{\sum_{2<f_k\le 5} \Gamma^X(f_k)}{\sum_{f_{inf}\le f_k\le 2} \Gamma^X(f_k)}$	(F19)
Frequency quotient AP	Ratio of energy content between 2-5 Hz and energy content at or below 2 Hz for AP coordinates	$\frac{\sum_{2<f_k\le 5} \Gamma^Y(f_k)}{\sum_{f_{inf}\le f_k\le 2} \Gamma^Y(f_k)}$	(F20)

Supplementary Table 6: Demographics and surface type of commonly misclassified individuals for PD vs. age-matched controls. In total, four subjects from the PD group and five subjects from the age-matched control group were commonly misclassified by the within static task only or active task only models. There was no consistent theme of factors, such as age, sex, or surface type, which seemed to influence these misclassifications.

PD vs. age-matched controls: **Static Tasks**

Group	Sex	Age	Surface
PD	Male	74	Pavement
Control	Female	59	Concrete
Control	Male	47	Concrete
PD	Male	64	Hardwood
Control	Female	54	Turf

PD vs. age-matched controls: **Active Tasks**

Group	Sex	Age	Surface
PD	Male	85	Hardwood
PD	Male	53	Hardwood
Control	Female	49	Turf
Control	Male	60	Hardwood
PD	Male	53	Carpet

Supplementary Table 7: Demographics and surface type of commonly misclassified individuals for PD faller vs. PD non-fallers. Again, there was not a consistent theme of factors, such as age or sex that influenced these misclassifications; however, most of the misclassifications were collected on the hardwood surface, which was at a local gym or in the individual's home. Further investigation as to how insole pressure sensors vary across data collection locations and surface types should be considered for future translational applications of insole plantar pressure sensors.

PD faller vs. PD non-faller: **Static Tasks**

Group	Sex	Age	Surface
Faller	Female	59	Hardwood
Faller	Male	61	Pavement
Faller	Male	74	Hardwood
Non-faller	Male	56	Hardwood
Non-faller	Male	63	Hardwood
Faller	Female	64	Hardwood
Non-faller	Female	74	Hardwood
Non-faller	Male	59	Hardwood
Faller	Female	73	Hardwood
Faller	Male	73	Hardwood
Faller	Female	69	Hardwood
Non-faller	Male	57	Hardwood

PD faller vs. PD non-faller: **Active Tasks**

Group	Sex	Age	Surface
Faller	Female	59	Hardwood
Non-faller	Female	67	Pavement
Faller	Male	73	Pavement
Non-faller	Male	62	Hardwood
Non-faller	Male	64	Hardwood
Faller	Male	67	Hardwood
Faller	Male	85	Hardwood
Non-faller	Female	74	Hardwood
Faller	Female	73	Hardwood
Faller	Male	73	Hardwood
Faller	Female	69	Hardwood
Non-faller	Female	70	Hardwood

PD faller vs. PD non-faller: **Static + Active Tasks**

Group	Sex	Age	Surface
Faller	Female	59	Hardwood
Faller	Male	61	Pavement
Faller	Male	73	Pavement
Faller	Male	74	Hardwood
Non-faller	Male	56	Hardwood
Non-faller	Female	74	Hardwood
Faller	Female	73	Hardwood
Faller	Male	73	Hardwood
Faller	Female	69	Hardwood

Supplementary Table 8: Common features for aged-matched controls vs. PD models with static tasks only, active tasks only, and static + active tasks

Common Features: Static Tasks Only

#	Type	Name
0	Avg	EO mean value ML
1	Avg	EO mean distance ML
2	Avg	EO root mean square ML
3	Avg	EO confidence ellipse area ML & AP

#	Type	Name
12	Avg	EO energy content 0.5 - 2 Hz power spectral density ML
13	Avg	EO energy content 0.5 - 2 Hz power spectral density AP
14	Avg	EO energy content above 2 Hz power spectral density ML
15	Avg	EO energy content above 2 Hz power spectral density AP

#	Type	Name
24	Avg	EC sway area / sec ML & AP
25	Avg	EC mean peak sway density
26	Avg	EC mean distance peak sway density
27	Avg	EC energy content 0.5 - 2 Hz pwer spectral density ML

4	Avg	EO mean velocity ML	16	Avg	EC mean distance ML	28	Avg	OF mean frequency ML
5	Avg	EO mean velocity AP	17	Avg	EC mean distance AP	29	Avg	OF mean frequency AP
6	Avg	EO sway area / sec ML & AP	18	Avg	EC mean distance radius	30	Avg	OF power frequency 50% power spectral density ML
7	Avg	EO phase plane parameter ML	19	Avg	EC maximal distance AP	31	Avg	OF energy content below 0.5 Hz power spectral density AP
8	Avg	EO length over area ML & AP	20	Avg	EC root mean square AP	32	Avg	OF frequency quotient power spectral density ML
9	Avg	EO peak velocity positive ML	21	Avg	EC coefficient sway direction ML & AP	34	Asym	EC zero crossing velocity AP
10	Avg	EO peak velocity positive AP	22	Avg	EC confidence ellipse area ML & AP			
11	Avg	EO mean peak sway density	23	Avg	EC mean velocity AP			

Common Features: Active Tasks Only

#	Type	Name	#	Type	Name	#	Type	Name
0	Avg	FR mean value AP	9	Avg	BO centroid frequency Power spectral density ML	18	Asym	BO phase plane parameter AP
1	Avg	FR length over area ML & AP	10	Avg	BO frequency quotient power spectral density ML	19	Asym	BO peak velocity positive AP
2	Avg	FR peak velocity neg vel. AP	11	Avg	BO frequency quotient power spectral density AP	20	Asym	BO peak velocity all vel. AP
3	Avg	FR peak velocity all vel. AP	12	Avg	GAIT mean value AP	21	Asym	BO frequency quotient power spectral density ML
4	Avg	BO mean value ML	13	Asym	FR zero crossing vel. ML	22	Asym	GAIT root mean square Radius
5	Avg	BO maximal distance AP	14	Asym	BO mean value ML	23	Asym	GAIT peak velocity pos AP
6	Avg	BO root mean square AP BO range AP	15	Asym	BO mean distance ML	24	Asym	GAIT frequency mode power spectral density ML
7	Avg	BO power frequency 95% power spectral density ML	16	Asym	BO maximal distance ML			
8	Avg	BO power frequency 95% power spectral density AP	17	Asym	BO maximal distance AP			

Common Features: Static + Active Tasks

#	Type	Name	#	Type	Name	#	Type	Name
0	Avg	EO mean value ML	30	Avg	EC mean velocity AP	60	Avg	FR phase plane parameter ML
1	Avg	EO mean distance ML	31	Avg	EC mean velocity ML & AP	61	Avg	FR phase plane parameter AP
2	Avg	EO mean distance AP	32	Avg	EC sway area per second ML & AP	62	Avg	FR LFS ML & AP
3	Avg	EO root mean square ML	33	Avg	EC phase plane parameter ML	63	Avg	FR peak velocity neg vel. ML
4	Avg	EO root mean square AP	34	Avg	EC zero crossing vel. ML	64	Avg	FR peak velocity all vel. ML
5	Avg	EO root mean square Radius	35	Avg	EC peak velocity pos vel. ML	65	Avg	FR peak velocity neg vel. AP
6	Avg	EO range ML	36	Avg	EC zero crossing vel. AP	66	Avg	FR peak velocity all vel. AP
7	Avg	EO confidence ellipse area ML & AP	37	Avg	EC peak velocity neg vel. AP	67	Avg	FR mean peak Sway Density
8	Avg	EO mean velocity ML	38	Avg	EC mean peak Sway Density	68	Avg	BO mean value ML
9	Avg	EO sway area per second ML & AP	39	Avg	EC total power power spectral density ML	69	Avg	BO maximal distance AP
10	Avg	EO phase plane parameter ML	40	Avg	EC total power power spectral density AP	70	Avg	BO maximal distance Radius
11	Avg	EO phase plane parameter AP	41	Avg	EC power frequency 95% power spectral density ML	71	Avg	BO range AP
12	Avg	EO length over area ML & AP	42	Avg	EC energy content below 0.5 Hz power spectral density ML	72	Avg	BO power frequency 95% power spectral density AP
13	Avg	EO peak velocity neg vel. AP	43	Avg	EC energy content below 0.5 Hz power spectral density AP	73	Avg	BO frequency quotient power spectral density AP
14	Avg	EO mean peak Sway Density	44	Avg	EC energy content 0.5-2 Hz Hz power spectral density ML	74	Avg	GAIT mean value AP
15	Avg	EO total power power spectral density ML	45	Avg	EC energy content above 2 Hz power spectral density ML	75	Asym	EO energy content 0.5-2 Hz Hz power spectral density ML
16	Avg	EO total power power spectral density AP	46	Avg	EC energy content above 2 Hz power spectral density AP	76	Asym	EC zero crossing vel. ML
17	Avg	EO energy content below 0.5 Hz power spectral density AP	47	Avg	EC frequency quotient power spectral density ML	77	Asym	EC zero crossing vel. AP
18	Avg	EO energy content 0.5-2 Hz Hz power spectral density ML	48	Avg	EC frequency quotient power spectral density AP	78	Asym	OF coefficient sway direction ML & AP
19	Avg	EO energy content 0.5-2 Hz Hz power spectral density AP	49	Avg	OF mean value ML	79	Asym	FR zero crossing vel. ML
20	Avg	EO energy content above 2 Hz power spectral density ML	50	Avg	OF mean distance ML	80	Asym	BO mean value ML
21	Avg	EO energy content above 2 Hz power spectral density AP	51	Avg	OF maximal distance ML	81	Asym	GAIT root mean square Radius
22	Avg	EC mean value ML	52	Avg	OF root mean square AP	82	Asym	GAIT planar deviation ML & AP

23	Avg	EC mean distance ML
24	Avg	EC mean distance Radius
25	Avg	EC maximal distance ML
26	Avg	EC maximal distance AP
27	Avg	EC coefficient sway direction ML & AP
28	Avg	EC confidence ellipse area ML & AP
29	Avg	EC mean velocity ML
53	Avg	OF range ML
54	Avg	OF power frequency 95% power spectral density ML
55	Avg	OF energy content below 0.5 Hz power spectral density AP
56	Avg	OF frequency quotient power spectral density ML
57	Avg	FR mean velocity ML
58	Avg	FR mean velocity AP
59	Avg	FR mean velocity ML & AP
83	Asym	GAIT principal sway direction ML & AP
84	Asym	GAIT zero crossing vel. ML
85	Asym	GAIT peak velocity positive AP
86	Asym	GAIT peak velocity all AP
87	Asym	GAIT frequency mode power spectral density ML

Supplementary Table 9: Common features for PD faller vs. PD non-faller models with static only tasks, active only tasks, and static + active tasks.

Common Features: Static Tasks Only

#	Type	Name
0	Avg	EC zero crossing vel. ML
1	Asym	EC mean distance AP
2	Asym	EC mean distance Radius
3	Asym	EC root mean square AP
4	Asym	EC range AP
5	Asym	EC range ML & AP
6	Asym	EC confidence ellipse area ML & AP
7	Asym	EC mean distance peak sway density
8	Asym	OF frequency mode power spectral density AP

Common Features: Active Tasks Only

#	Type	Name
0	Avg	FR length over area ML & AP
1	Avg	GAIT mean value AP
2	Avg	GAIT zero crossing vel. AP
3	Avg	GAIT total power power spectral density ML
4	Avg	GAIT energy content below 0.5 Hz power spectral density ML
5	Avg	GAIT energy content 0.5-2 Hz power spectral density AP
6	Asym	FR confidence ellipse area ML & AP
7	Asym	BO power frequency 50% power spectral density AP
8	Asym	GAIT sway area per second ML & AP
9	Asym	GAIT energy content above 2 Hz power spectral density ML

Common Features: Static + Active Tasks

#	Type	Name
0	Avg	EC zero crossing velocity ML
1	Avg	EC zero crossing velocity AP
2	Avg	OF power frequency 50% power spectral density ML
3	Avg	GAIT mean value AP
4	Avg	GAIT zero crossing velocity ML
5	Avg	GAIT zero crossing velocity AP
6	Avg	GAIT total power power spectral density ML
7	Avg	GAIT energy content below 0.5Hz power spectral density ML
8	Avg	GAIT energy content 0.5-2 Hz power spectral density ML
9	Avg	EC range AP
10	Asym	EC confidence ellipse area ML & AP
11	Asym	OF frequency mode power spectral density AP
12	Asym	FR length over area ML & AP
13	Asym	FR frequency dispersion power spectral density ML
14	Asym	BO power frequency 50% power spectral density AP
15	Asym	GAIT sway area per second ML & AP
16	Asym	GAIT energy content above 2 Hz power spectral density ML
