# Supplementary material for: Distinguishing features of Parkinson’s disease fallers based on wireless insole plantar pressure monitoring
Source: NPJ Parkinsons Dis. 2024 Mar 19;10:67. doi: 10.1038/s41531-024-00678-2 (PMC10951221; doi:10.1038/s41531-024-00678-2)
Supplement: Supplementary file 2 — Reporting Summary [file 41531_2024_678_MOESM2_ESM.pdf]

nature portfolio

Corresponding author(s): Matthew D. Johnson

Last updated by author(s): 12/6/2023

Reporting Summary

Nature Portfolio wishes to improve the reproducibility of the work that we publish. This form provides structure for consistency and transparency in reporting. For further information on Nature Portfolio policies, see our Editorial Policies and the Editorial Policy Checklist.

Statistics

For all statistical analyses, confirm that the following items are present in the figure legend, table legend, main text, or Methods section.

n/a	Confirmed	
☐	☒	The exact sample size (*n*) for each experimental group/condition, given as a discrete number and unit of measurement
☐	☒	A statement on whether measurements were taken from distinct samples or whether the same sample was measured repeatedly
☐	☒	The statistical test(s) used AND whether they are one- or two-sided<br>*Only common tests should be described solely by name; describe more complex techniques in the Methods section.*
☐	☒	A description of all covariates tested
☐	☒	A description of any assumptions or corrections, such as tests of normality and adjustment for multiple comparisons
☐	☒	A full description of the statistical parameters including central tendency (e.g. means) or other basic estimates (e.g. regression coefficient) AND variation (e.g. standard deviation) or associated estimates of uncertainty (e.g. confidence intervals)
☐	☒	For null hypothesis testing, the test statistic (e.g. *F*, *t*, *r*) with confidence intervals, effect sizes, degrees of freedom and *P* value noted<br>*Give P values as exact values whenever suitable.*
☒	☐	For Bayesian analysis, information on the choice of priors and Markov chain Monte Carlo settings
☒	☐	For hierarchical and complex designs, identification of the appropriate level for tests and full reporting of outcomes
☒	☐	Estimates of effect sizes (e.g. Cohen's *d*, Pearson's *r*), indicating how they were calculated

Our web collection on statistics for biologists contains articles on many of the points above.

Software and code

Policy information about availability of computer code

Data collection	During all tasks, plantar pressure data was collected using insole plantar pressure sensors (PRISM, RetiSense, India) in which the sensor unit streamed data to a mobile device using the freely available RetiSense Insight App.
Data analysis	The back-end of the RetiSense Insight App software averaged the data from the 100 pressure sensors into eight groups, which were determined by anatomically relevant locations.

For manuscripts utilizing custom algorithms or software that are central to the research but not yet described in published literature, software must be made available to editors and reviewers. We strongly encourage code deposition in a community repository (e.g. GitHub). See the Nature Portfolio guidelines for submitting code & software for further information.

Data

Policy information about availability of data

All manuscripts must include a data availability statement. This statement should provide the following information, where applicable:

- Accession codes, unique identifiers, or web links for publicly available datasets
- A description of any restrictions on data availability
- For clinical datasets or third party data, please ensure that the statement adheres to our policy

Data and code are available in our github repository: https://github.umn.edu/NRTL/InsoleML.

Research involving human participants, their data, or biological material

Policy information about studies with human participants or human data. See also policy information about sex, gender (identity/presentation), and sexual orientation and race, ethnicity and racism.

Reporting on sex and gender	All participants completed a questionnaire with self-reported information on their sex. Contributions of sex as a biological variable to misclassifications were analyzed (Tables S6, S7).
Reporting on race, ethnicity, or other socially relevant groupings	All participants completed a questionnaire with self-reported information on their race.
Population characteristics	All participants completed a questionnaire with self-reported information on their age. Contributions of age to misclassifications were analyzed (Tables S6, S7).
Recruitment	Participants were recruited through public events and Parkinson disease community gatherings.
Ethics oversight	The study was approved by the University of Minnesota Institutional Review Board (IRB Study Number: STUDY00013580).

Note that full information on the approval of the study protocol must also be provided in the manuscript.

Field-specific reporting

Please select the one below that is the best fit for your research. If you are not sure, read the appropriate sections before making your selection.

☒ Life sciences ☐ Behavioural & social sciences ☐ Ecological, evolutionary & environmental sciences

For a reference copy of the document with all sections, see nature.com/documents/nr-reporting-summary-flat.pdf

Life sciences study design

All studies must disclose on these points even when the disclosure is negative.

Sample size	We determined sample sizes based on previous studies investigating center of pressure differences in people with Parkinson's disease. In our study, we greatly surpassed the sample sizes used in those previous studies.
Data exclusions	Data were not excluded from the analysis.
Replication	We used five distinct models and five-fold cross-validation to increase the scientific rigor of the results and interpretation. At each stage of the sequential feature selection process, the model chose the best feature to add based on the five-fold cross-validation F1 score of the model. This was repeated until all features had been sequentially added to the model and assessed for the five-fold cross-validation F1 score.
Randomization	Participants were assigned to groups based on their self-reported survey responses (age, diagnosis of PD, history of falls within the past year).
Blinding	Study staff were not privy to the survey results upon performing the data collection tasks, albeit some context was inherently present during data collection such as collecting data at a Parkinson's community gathering. After assignment to groups based on the survey results, subjects were de-identified during the data processing, feature selection, and machine learning model analysis.

Reporting for specific materials, systems and methods

We require information from authors about some types of materials, experimental systems and methods used in many studies. Here, indicate whether each material, system or method listed is relevant to your study. If you are not sure if a list item applies to your research, read the appropriate section before selecting a response.

Materials & experimental systems

n/a	Involved in the study
☒	☐ Antibodies
☒	☐ Eukaryotic cell lines
☒	☐ Palaeontology and archaeology
☒	☐ Animals and other organisms
☒	☐ Clinical data
☒	☐ Dual use research of concern
☒	☐ Plants

Methods

n/a	Involved in the study
☒	☐ ChIP-seq
☒	☐ Flow cytometry
☒	☐ MRI-based neuroimaging

Plants

Seed stocks	N/A
Novel plant genotypes	N/A
Authentication	N/A
